# The Job Perception Inventory: considering human factors and needs in the design of human–AI work

**DOI:** 10.3389/fpsyg.2023.1128945

**Published:** 2023-05-23

**Authors:** Sophie Berretta, Alina Tausch, Corinna Peifer, Annette Kluge

**Affiliations:** ^1^Department of Work and Organizational Psychology, Ruhr University Bochum, Bochum, Germany; ^2^Institute of Psychology I, University of Lübeck, Lübeck, Germany

**Keywords:** work psychology, human-centered AI, job identity, wellbeing, motivation, survey inventory, new work, validation

## Abstract

**Introduction:**

Artificial intelligence (AI) is seen as a driver of change, especially in the context of business, due to its progressive development and increasing connectivity in operational practice. Although it changes businesses and organizations vastly, the impact of AI implementation on human workers with their needs, skills, and job identity is less considered in the development and implementation process. Focusing on humans, however, enables unlocking synergies as well as desirable individual and organizational outcomes.

**Methods:**

The objective of the present study is (a) to develop a survey-based inventory from the literature on work research and b) a first validation with employees encountering an AI application. The Job Perception Inventory (JOPI) functions as a work-analytical tool to support the human-centered implementation and application of intelligent technologies. It is composed of established and self-developed scales, measuring four sections of work characteristics, job identity, perception of the workplace, and the evaluation of the introduced AI.

**Results:**

Overall, the results from the first study from a series of studies presented in this article indicate a coherent survey inventory with reliable scales that can now be used for AI implementation projects.

**Discussion:**

Finally, the need and relevance of the JOPI are discussed against the background of the manufacturing industry.

## 1. Introduction

Remaining competitive in the manufacturing industry requires faster and more effective production, with a low error rate and minimal costs (Helmold, 2021). To meet these requirements, the use of automation and other technologies proved useful in the past. Nowadays, strong interest appears especially in AI technologies, which have a broader range of usage and application compared to automation (Ribeiro et al., 2021). AI technologies have the overarching goal of emulating human capabilities and cognitions, such as learning, interacting, problem-solving, decision-making, persuading, and acting (e.g., Huang et al., 2019; Rai et al., 2019; Dellermann et al., 2021). Thus, AI is seen as a driver of productivity and progress, anticipating and predicting major societal and work-related changes (e.g., Brynjolfsson and McAfee, 2014; MacCrory et al., 2014).

However, these assumptions are not new. AI technologies have a history of more than 70 years, during which it has already been predicted several times that AI will fundamentally change the future of the working world (Zhang and Lu, 2021). What is missing most in this discourse, both in the past and today, is how employees can continue to work in an AI-induced workplace that is conducive to motivation, wellbeing, and identity (Parker et al., 2017). If we are not able to answer this question in research, work design, and as a society, the advantages of AI usage will never come to their full potential, and we will run into an AI crisis. In this study, we, therefore, concentrate on the human side, understanding AI technologies as a working feature that affects employee experience and has to be designed for, not apart from, human work (places).

In the past, principles of design, implementation, and application of technical systems often focused on the technology itself and the associated strengths without considering the consequences or strengths of the human part (Kluge et al., 2021). That needs to change. A useful approach, which attempts to unite the strengths of an AI system with those of the human, is human–AI teaming (Huchler, 2022; Mirbabaie et al., 2022). Human–AI teaming describes the cooperation between the human and technical parts, which is characterized by mutual support, collaboration, transparent thought and action processes, and a common situational understanding (Kluge et al., 2021). This collaboration makes it possible to compensate for errors and to achieve better results (Jarrahi, 2018; Dubey et al., 2020; Kluge et al., 2021) because the capabilities and strengths of an AI system and a human complement each other ideally (Huchler, 2022): On the one hand, the strengths of AI can be identified in rational, analytical decision-making and the achievement of quantitative goals (Parry et al., 2016). AI systems have been outperforming humans in terms of computational capabilities for several years. Humans, on the other hand, can adapt flexibly to different contexts and perform better in evaluating and achieving qualitative, subjective goals (Jarrahi, 2018). At the same time, humans can draw on knowledge and experience, which is partly subconsciously and intuitively anchored, making them a unique, irreplaceable resource (Buchanan and Connell, 2006). Accordingly, the unique strengths of humans and AI can be synergistically potentiated through collaboration, leading to the desired goals of improved productivity and performance. Moreover, in addition to beneficial organizational goals, positive effects occur on the part of the individual. As AI systems take over repetitive, monotonous tasks, there is more time for humans to turn to identity-forming tasks (Jarrahi, 2018; Yang and Siau, 2018). Consequently, motivation and competencies within the human part can be promoted (Hughes et al., 2019).

Designing a workplace that enables the described teaming, flexible and adapted to the needs of humans, is the vision of work that we aim to shape. However, the reality is still far away from this: First, most of the AI systems in use belong to the class of narrow AI, which includes restricted self-learning approaches (Batin et al., 2017; Hole and Ahmad, 2021). Systems based on narrow AI are limited to very specific, bounded tasks so a flexible, adaptable teaming approach cannot be implemented with narrow AI from a purely technical point of view. Rather, it requires at least general AI, or super-intelligent AI, whose self-learning capacities are comparable to human intelligence and thus enable teaming approaches in the workplace (Batin et al., 2017; Scott et al., 2022). In addition, the implementation of human–AI teaming is also hindered by the fact that the impact of AI usage is often not elaborated in advance, and thus the tasks of the AI and those of the human are not optimally aligned with each other (Li and Lee, 2022). The most common approach is to develop and introduce an intelligent technology without considering the workplace and the tasks it contains beforehand (Ötting, 2020). Such an approach hinders a human-centered implementation and especially real collaboration between humans and AI (Al Ali and Badi, 2022; Tegtmeier et al., 2022). To overcome these barriers and enable a teaming approach, we developed an instrument to implement AI with a focus on humans with their needs, motives, and capabilities. Basing an AI implementation on this unlocks synergies in the workplace (Wilkens et al., 2020; Kluge et al., 2021) and promotes wellbeing, motivation, job identity, and in turn performance and organizational outcomes (Parker and Grote, 2020; Huchler, 2022).

To realize such a human-centered implementation strategy in organizations, they need understandable, easy-to-use, and scientifically founded methods and tools that guide them to human–AI teaming. Current approaches to accompany a human-centered AI implementation are mostly on a theory-based level (e.g., Degen and Ntoa, 2021; Wilkens et al., 2021; Tjondronegoro et al., 2022). However, a practically applied method that captures relevant aspects in the context of AI introductions and allows us to derive a human-centered implementation strategy in terms of a human–AI teaming workplace does not exist. Therefore, this study describes the newly developed Job Perception Inventory (JOPI) as a tool for the practical application of human-friendly AI implementation. The JOPI aims to capture relevant work characteristics, job identity, and the perception of the workplace, as well as the evaluation of working with AI to derive a human-centered implementation strategy, for human–AI teaming. It is tested for reliability and validity and results from the first sample are presented. To underline the practical implementation of the JOPI, we transfer our insights to the manufacturing industry and advise on how to use the instrument there to create human–AI teaming workplaces in a production setting.

## 2. Need and the idea of the JOPI

The relevance of an inventory that contributes to a human-centered technology implementation is mainly based on past, backlashing experiences in the context of automation. There, the implementation and subsequent work design often focused primarily on the technical system with its features and benefits, while the unique capabilities of employees became secondary (Nahavandi, 2019). The aim was to automate as many activities as possible and thus replace the supposedly biggest weak point in the system—the human being. Ironically, since the 1970s, evidence has instead shown a negative correlation between economic productivity and intensive investments in digital technologies (Brynjolfsson, 1993). This productivity paradox means that despite the increased costs of acquiring such systems, there is a counterproductive slowdown in performance, which obviously has negative consequences for organizations—and is potentially due to not involving the human part in the redesign of work. It turns out that about half of the deceleration can be explained by (decreasing) employees' productivity (Gordon, 2018), who in addition experienced further undesirable side effects: experiencing monotony (Ralph et al., 2017), loss of skills (Frank and Kluge, 2019) with simultaneously increasing demands (Rieth and Hagemann, 2021), reductions in attention (Parasuraman et al., 1993), the unreflective acceptance of decision proposals by technology (Dalcher, 2007), and too much or too little trust in automation (Merritt et al., 2019). Reduced wellbeing and the fear of being completely replaced at the workplace resulted and persist (Vorobeva et al., 2022). These consequences for employees received little attention until it became apparent how serious they were for individuals and organizations. As a solely robot-driven production failed and the automation focus demonstrably slowed down productivity in manufacturing factories, even Tesla's high-profile CEO Elon Musk acknowledged that “Humans are underrated” (Musk, 2018). Now, the level of automation in the factories was scaled back in favor of better organizational performance (CBS News, 2018).

To ensure to use the lessons learned from the past, we argue to focus on humans to align work design and technology with their needs. This approach is inherent to the project's context HUMAINE in which JOPI is developed. In cooperation with practice and research partners from occupational science, business psychology, engineering, and several further disciplines, an integrative approach to human-centered AI development, implementation, and use is pursued and applied in multiple pilot projects. The focus is on human-centeredness and ensuring the occupational health and safety of affected employees. The insights gained will lead to the establishment of a competence center that provides development guidelines, implementation strategies, and best practice models for practical use. The work in the project is based on sociotechnical theory, and its idea that the complex human system is inseparably linked to technological elements in the workplace (Trist and Bamforth, 1951; Maguire, 2014; Gabriel et al., 2022). Changes on the one side inevitably entail changes on the other side (Cherns, 1976). Due to this close relationship, it is necessary to consider the social and technological components together and finally to carry out a joint optimization (Vecchio and Appelbaum, 1995). Through a joint optimization of the technical and social system, it is then possible to benefit from improved performance without disregarding human needs (Hamilton et al., 2008). An important tool for uncovering sociotechnical components and developing workplaces in the direction of joint optimization is psychological work analysis, in which the work tasks and environment are systematically captured and evaluated, as well as their influence on employees (Schaper, 2014). Various methods can be used to determine these influencing factors, including work observations, questionnaires, interviews, or simulations (Schaper, 2014).

The JOPI can be described as a tool of psychological work design from the perspective of the workplace holder, consisting of different questionnaire procedures for analyzing motivation, wellbeing, and job identity in workplaces confronted with AI implementations.

For the development of the JOPI, established scales as well as new scales are used. We first reviewed the current literature on work characteristics, job identity, perceptual factors, and the influence of technology under the implementation of AI in the workplace. As questionnaires are a popular method in psychology that are easy to use (Moosbrugger and Kelava, 2020; Krumm et al., 2021), and offer a standardized way of examining people's perception of their job and tasks, the JOPI is also designed as a questionnaire inventory. Existing instruments for capturing work characteristics, job identity, and perceptions were examined for (a) relevant items and (b) an appropriate format. Wherever suitable, those previously published and validated scales were used, supplemented with newly developed or modified items and scales. Those scales were developed following the internal or factor-analytical construction strategy. In this theory-based construction principle, the goal is to identify groups of items, or subscales, of a construct that are distinct from other subscales of the same construct (Jonkisz et al., 2012). The items of these subscales are derived from the hypothesized behavioral dimensions of the defined construct and previous literature. We then conducted a validation study for the instrument with a sample being confronted with the approaching implementation of an AI tool—in this case, speech therapists. With the help of the generated results, the intended factor structure with its subscales can be statistically verified (Jonkisz et al., 2012). According to this construction principle, items of relevant constructs regarding the introduction of AI were derived and then factor-analyzed. In this study, we show the JOPI and the corresponding results of this validation—and then transfer these insights to the manufacturing industry, which we see as one highly relevant field of application for the JOPI.

All in all, the objective of the JOPI is to fulfill the important function of work analysis to identify design and optimization potentials before, during, and after AI implementation. Based on this analysis, JOPI contributes to personality- and health-promoting work design in human–AI teaming. Focusing on humans instead of AI functionality forms the basis for adapting technologies, their use, and the organization of work to human needs and capabilities. The composition is modularly changeable and can be adapted to the respective context of an investigation. As a result, the JOPI is not limited to one industry or one application context but is intended to be used across various industries.

## 3. Conceptualization and use of the JOPI

The intended application procedure is to use the JOPI multiple times, especially before AI implementation and ideally during the developmental process. This ensures that organizations know about the current work characteristics (Section 1) and thus resources and demands of the job without an AI, about what complies with the identity of a certain job (Section 2), to ensure that these parts are not endangered by AI, and how employees experience their work (Section 3) cognitively and affectively. Additional applications of the inventory should follow during and after AI implementation. This last step is important not only to see how a workplace and its perception have changed due to AI usage but also to evaluate the AI and its suitability (Section 4).

### 3.1. Work characteristics

The way a job is perceived by employees depends on work characteristics (Harter et al., 2010). They include the social and organizational environment as well as specific attributes of everyday work tasks (Stegmann et al., 2010). Work characteristics and their impact have a long history of research (Parker et al., 2017), which highlights the importance of considering them in designing a productive and satisfactory work environment. From this research history, a total of five perspectives concerning work characteristics and their effects can be identified. These include, for example, the socio-technical theory of Trist and Bamforth (1951), and also other well-known models such as the Job-Demands-Resources Model (JD-R; Demerouti et al., 2001) or the Job Characteristics Model (JCM; Hackman and Oldham, 1975). The JD-R model mainly focuses on the dependency between demands and resources, whereas the JCM focuses more on job characteristics and related psychological states, such as satisfaction. As an extension to these perspectives, which usually focus on one aspect of work design at a time, Parker et al. (2017) argue for a more integrative and contemporary model that combines all previous perspectives in this field. That is why she developed the idea of SMART work being stimulating, fostering mastery and agency, providing relational aspects, and posing tolerable demands on the workplace holders (Parker, 2014; Parker et al., 2017). Together with Gudela Grote, Sharon Parker also transferred the idea of key work characteristics to the context of technology introduction (Table 1; Parker and Grote, 2020), because in the case of changes due to technologies, a good design of work characteristics can significantly influence the perception of this change. Work characteristics are a “powerful vehicle” (Parker et al., 2017, p. 415) and can buffer potential negative effects as well as strengthen possible positive effects. Thus, it is important for AI-induced changes to take into account work characteristics and to design them beyond the change in such a way that possible negative effects caused by the implementation are mitigated and potential positive effects are reinforced from the beginning.

**Table 1:** Possible effects on relevant work characteristics under the use of AI.

**Work characteristic**	**Associated outcome(s)**	**Potential positive development**	**Potential negative development**
Autonomy	Motivation (Dysvik and Kuvaas, 2011), performance (Langfred and Moye, 2004), creativity (d'Inverno and Luck, 2012), and proactivity (Kim et al., 2009)	Possibility for autonomous action could increase, as access and storage of knowledge occurs internet-based (Sundararajan, 2016).	AI takes over decision-making, which would reduce perceived autonomy (Charlwood and Guenole, 2022).
Variety of requirements	Job satisfaction (Humphrey et al., 2007)	AI takes over dangerous and mundane tasks so that the human can invest more time in meaningful, challenging tasks (Jarrahi, 2018).	AI takes over a large part of the tasks, turning a previously active employee into a passive controller (Rieth and Hagemann, 2021).
Job Feedback (includes role clarity and task identity)	Maintenance of skills (Arco and Du Toit, 2006), performance (Vigoda-Gadot and Angert, 2007), and motivation (Sultan, 2012)	AI can provide individualized feedback and thus contribute to role clarity (Jain et al., 2022).	The use of AI and the accompanying changes at work can lead to role insecurity (Shepherd, 2006).
Social and relational	Affective work outcomes such as satisfaction (Santos et al., 2016)	AI can improve facilitated, location-independent exchange between employees (Neeley and Leonardi, 2018).	Difficulty of use or lack of trust in AI can hinder exchange between employees (e.g., Möhlmann and Zalmanson, 2017).
Work demands	Changes the demands that exist for employees (Beer and Mulder, 2020)	AI takes over monotonous tasks, leaving more stimulating and cognitively demanding tasks for humans (Yang and Siau, 2018).	AI takes over a large part of the tasks employees have to monitor, which is perceived as stressful and tiring (e.g., Wisskirchen et al., 2017).

Overall, work characteristics and job design have profound effects on employees' affective and performance components (e.g., Noblet, 2003; Lawson et al., 2009; Parker et al., 2017). The influence can be both positive, by strengthening existing resources, and negative, by transforming resources into demands (see Table 1).

For a human-centered AI integration into the workplace, it is important to determine the current state of work characteristics to derive an implementation strategy and to positively influence their development (Tegtmeier et al., 2022). Therefore, the first section of the JOPI aims to capture relevant work characteristics concerning the implementation of smart technologies. Within the five perspectives that have evolved regarding the research of work characteristics, various survey instruments have also been developed to measure them. For example, following the JCM, the Job Diagnostic Survey (JDS; Hackman and Oldham, 1975) was developed to measure a small sample of job characteristics and possible psychological states. Similar to the perspectives on work characteristics, the instruments lack a holistic, integrative approach, so that usually only a small, specific range is surveyed. The Work Design Questionnaire (WDQ; Morgeson and Humphrey, 2006) is the first attempt to pursue a broader approach in which, besides a wide range of work characteristics, task-spanning characteristics are also surveyed (Parker et al., 2017). However, for our needs, the WDQ collects too many work characteristics that are not directly related to the implementation of AI, such as physical ergonomics, whereas other important characteristics, such as psychological demands, are not assessed. Hence, we decided to develop precise items for the examination of relevant work characteristics and if possible, to orientate ourselves on the well-established WDQ scale. The aim was to cover the five core characteristics identified by Parker and Grote (2020), as these were shown to be both important and influenceable in the context of technology introductions.

Consequently, we measure perceived *autonomy, variety of requirements* including a variety of demands and information processing, and *changes in work demands* including psychological demands and technology use with a total of 12 items (see Table 2). Information regarding one's role (*job feedback*) and interaction opportunities (*social and relational work characteristics*) in the workplace are collected in other sections of the JOPI.

**Table 2:** Items of the JOPI to assess relevant work characteristics.

**No**.	**Item**	**Polarity**	**Scale**
1	At work, I have a lot of freedom in terms of planning and performing my tasks.	+	AT
2	I can decide a lot of things independently at work.	+	AT
3	The way I manage my work tasks is up to me.	+	AT
4	My work allows me to bring a variety of different skills to the table.	+	VOD
5	I do a lot of different tasks at work that challenge me in different ways.	+	VOD
6	I have to simultaneously look at a lot of information in my work.	+	IP
7	My work allows me to process incoming information one by one.	–	IP
8	Part of my job is to use technologies.	+	TU
9	In my work, I do not rely on the use of technology.	–	TU
10	I work under high time pressure.	+	PD
11	My work is emotionally demanding.	+	PD
12	In my job, I have to manage a large workload.	+	PD

The items were conducted in German on a 6-point Likert scale from “strongly disagree” to “strongly agree”. AT, autonomy; VOD, variety of demands; IP, information processing; TU, technology use; PD, psychological demands.

The items for assessing perceived *autonomy, variety of demands*, and *information processing* were re-developed, but based on the WDQ. The redesign was necessary to make the items more concise and to shorten the number slightly. For example, regarding *autonomy*, the WDQ differentiates between autonomy in scheduling (e.g.,: “The job allows me to decide on the order in which things are done on the job.”), decision-making (e.g.,: “The job provides me with significant autonomy in making decisions.”), and methods (e.g.,: “The job allows me to make decisions about what methods I use to complete my work.”). Thus, we have tried to capture *autonomy* on a more general level with fewer items for an economic inventory. Concerning the *variety of demands* and *information processing*, the main goal of the redesign was also to reduce the number of items and to keep the inventory economical despite the large number of scales in the JOPI. Both subscales are collected with only two from the original four items.

For assessing *technology use*, new items were needed as the wording of the original items in the WDQ was partly outdated (e.g.,: “The job involves the use of a variety of different equipment.”), especially in the German language.

Finally*, psychological demands* are not assessed in the WDQ. We have additionally developed these items because changes in work demands are to be expected due to the introduction of technology and these also include psychological demands (Parker and Grote, 2020). Otherwise, without such items, only changes in cognitive demands are measured by the subscales of a *variety of demands* and *information processing*.

With this presented scale consisting of new and edited items shown in Table 2, we hope to determine important work characteristics. In this way, it can be assessed in an economic way whether the introduced technology is more likely to become a resource or a burden in the workplace, whereupon appropriate steps can be derived.

To sum up, the section about work characteristics in the JOPI provides information about job design and requirements, as well as important clues about work resources, demands, and job identity (Braine and Roodt, 2011). The findings obtained can be used to derive a human-centered implementation strategy for AI.

### 3.2. Job identity

Identity is understood as the answer to the question “Who am I” (Carter and Grover, 2015) and functions as a mechanism to navigate social reality (Endacott, 2021). Depending on the social context, people develop several identities (Scott et al., 1998; Ramarajan, 2014). Even the work context is an environment where people develop an identity, which is a very central identity, as people usually spend a large part of their lives at work (Crocetti et al., 2014; Sadeghian and Hassenzahl, 2022). The so-called job identity can be understood as an organizing structure, consisting of various work-related norms, values, and resources (Scott et al., 1998; Reay et al., 2017), which influences how a person feels and behaves at work (Cerulo, 1997; Leonardi, 2015). The work-related norms, values, and resources comprise “core beliefs or assumptions, attitudes, preferences, decisional premises, gestures [and] habits” (Scott et al., 1998, p. 303). These are encountered by persons during their work and then coalesce into a relatively stable job identity that serves as an anchored repertoire of various practices and resources on which people rely while acting (Scott and Myers, 2010). The process of identity formation is thereby an iterative process that is shaped primarily by interaction with others (Crocetti et al., 2008). Through the reaction of others to one's own identity, which can be expressed through communication strategies (Watson and Watson, 2012), decision-making (Evans et al., 2004), and the expression of emotions (Rafaeli and Sutton, 1989), one's own identity and the values it contains can be evaluated and slightly modified. However, such a change in one's own job identity is feasible only if it is intrinsically motivated and the modification is not too extensive (Soenens and Vansteenkiste, 2011; Choudhry et al., 2017). Apart from that, identity is a robust construct that reacts to changes with rejection (Reay et al., 2017), especially if these changes are externally induced (Soenens and Vansteenkiste, 2011). Implementing AI is thus an externally induced change that can result in new work practices and consequently require a behavior change, which indirectly influences job identity (Leonardi and Bailey, 2008). If these changes due to AI use are too extensive and affect identity-forming norms, values, and resources (core aspects) of job identity, they are perceived as a threat (Petriglieri, 2011). A loss of self-esteem and lower satisfaction may result (Petriglieri, 2011), as well as a rejection of human–AI teaming (Mirbabaie et al., 2022). Accordingly, job identity is an important influencing variable that should be considered in the context of workplace changes.

To prevent the rejection of implemented AI, the job identity needs to be assessed holistically so that an implementation strategy can be developed that strengthens, rather than threatens, the job identity. That is why we use different measures to approach different sources of information on peoples' job identities. As a starting point, it is useful to ask about the motives for choosing a profession because such motives may be a predictor for job identity (Ölmez-Çaglar, 2022). For example, the choice of occupation needs to consider what kind of work corresponds to one's field of interest and to which occupational group one should belong in future. Thus, the motives of choosing a profession already allow insights into personal goals and interests and, at the same time, preferences for group affiliation (Crocetti et al., 2018), which could provide important clues for job identity. A scale was developed in which participants have to rank between 12 different motives for choosing a profession (see Table 3). The motives presented are supposed to be sorted by the participants according to relevance to be able to draw initial conclusions about the job identity.

**Table 3:** Motives for choosing a profession.

**No**.	**Item**
1	Opportunity to earn a high salary
2	Number of hours worked per week
3	Opportunity for career advancement
4	Working with people
5	Helping people
6	Variety at work
7	Opportunity to take on responsibility
8	Self-realization
9	Interest in work content
10	Expectation of others
11	(Family) tradition
12	Compatibility with family

The items were used in German. The most important item for the career choice should be placed at the top and the least important item at the bottom.

As a second variable to assess job identity holistically, it is useful to assess the activities within a workplace to deduce the core aspects of job identity. As identity is not an abstract construct but reveals itself directly in actions and interactions (Leonardi, 2015), activities at the workplace should be a valid indicator of the core aspect of job identification. The objective of this dimension is to ascertain what kind of activity a person identifies with within their workplace, for example, with more creative activities or with social work tasks (task-based identity). In this, we expect to find inter-individual differences as well as certain patterns for different jobs or branches that are similar. After all, a job in any industry involves the performance of various activities, but only a part of these belong to job identity (Thompson et al., 2018). These tasks, which are part of the job identity, need to be figured out to protect them from upcoming changes and technology introductions. A total of 11 different job profiles were extracted from the requirement profiles of the Fleishman Job Analysis System (F-JAS; Kleinmann et al., 2010) with which a person can identify in the workplace. These include (1) creative activities, (2) activities that promote learning and curiosity, (3) activities that involve solving complex problems, (4) activities that help other people, (5) activities carried out with others, (6) activities that involve hands-on work, (7) activities involving the pursuit of justice, (8) activities involving responsibility, (9) activities that are low (cognitively) demanding, (10) activities that involve high prestige, and (11) activities that require a high level of expertise. We then formulated three items for each of those activities, at least one of which is negatively poled, to capture core activities for the employees as precisely as possible. These resulted in the task-based identity scale (TBIS; Table 4).

**Table 4:** Items for measuring task identity.

**No**.	**Item**	**Polarity**	**Scale**
1	Being creative is very important to me in my professional life.	+	CA
2	If possible, I try to avoid creative tasks in my professional life.	–	CA
3	When I decided on my current profession, creativity played a big role.	+	CA
4	Learning something new excites me.	+	LC
5	I am not very interested in acquiring new job-related knowledge.	–	LC
6	One of the main reasons for choosing my current job is that my curiosity is encouraged there.	+	LC
7	I like to solve very complex problems.	+	CP
8	In my everyday work, I prefer tasks that can be solved without much thinking.	–	CP
9	I chose my current job primarily to deal with complex problems.	+	CP
10	Supporting others through my work is very important to me in my professional life.	+	HP
11	I consciously chose my current job to be able to help others.	+	HP
12	Helping others does not play a big role in my professional life.	–	HP
13	The most important thing for me in my professional life is to interact with other people.	+	SC
14	I chose my current job because of the social contacts.	+	SC
15	I prefer working alone.	–	SC
16	I prefer to work with my head instead of my hands.	–	HO
17	I chose my current job because it allows me to work practically.	+	HO
18	It is important to me to be able to “lend a hand” in my profession.	+	HO
19	I want to use my work to actively fight injustice.	+	JA
20	I chose my current job to be able to reduce injustice.	+	JA
21	I don't think much about the extent to which my work affects the (un)fair treatment of people.	–	JA
22	It is important to me to promote the wellbeing or safety of others through my work.	+	AR
23	I chose my current job to be able to contribute to society.	+	AR
24	The social contribution of my work is not important to me.	–	AR
25	I fulfill my professional tasks without thinking long and hard about the reasons behind them.	+	LD
26	I chose my current job mainly because of the consistent tasks.	+	LD
27	I like to solve complicated and thought-provoking tasks.	–	LD
28	I like being admired by others for my work.	+	AP
29	I chose my current job because it comes with a high reputation.	+	AP
30	I don't care what others think of my work.	–	AP
31	It is important to me to use my competences in my everyday professional life.	+	AE
32	I consciously chose a job where I can contribute my expertise.	+	AE
33	Almost anyone can do my work without much training.	–	AE

The items were originally formulated in German. CA, creative activities; LC, activities that promote learning and curiosity; CP, activities that involve solving complex problems; HP, activities that help other people; SC, activities carried out with others; HO, activities that involve hands-on work; JA, activities involving the pursuit of justice; AR, activities involving responsibility; LD, activities that are low (cognitively) demanding; AP, activities that involve high prestige; AE, activities that require a high level of expertise.

Additionally, it is appropriate to survey other variables besides the core aspects of job identity. This is important to get a full picture of peoples' identity not only from a qualitative content point of view but also quantitatively, i.e., its extent, and in relation to different levels of their job. Here, the person's *self-concept* comes into play, which is closely linked to identity (Markus and Wurf, 1987) and is therefore also captured in the JOPI. The self-concept represents the cognitive identity component which arises via generalization processes from situational self-assessments (Martschinke, 2011). Thereby, a stable self-concept is associated with successful identity development and correlates with openness to new things (Crocetti et al., 2008). Thus, the stability of the self-concept could provide indications of openness to new things in the workplace, such as an AI introduction (Rossi et al., 2018; Bhargava et al., 2021; Mirbabaie et al., 2022). Using the Utrecht Management Of Identity Commitment Scale (U-MICS; Crocetti et al., 2010) in our JOPI allows further conclusions regarding the stability of the self-concept (Crocetti et al., 2008). In this scale, a distinction is made between three different stages: *Commitment, in-depth exploration*, and *reconsideration of commitment*. *Commitment* is characterized by feelings of security and self-confidence that result from enduring choices at work (Crocetti et al., 2010; e.g., “My job gives me security for the future.”). In *in-depth exploration* stage, the individual is continuously reflecting on the professional situation and seeking to validate it with additional information (Crocetti et al., 2010; e.g., “I often reflect my job.”). When *reconsidering the commitment*, the current work is perceived as unsatisfactory, and reorientation often occurs (Crocetti et al., 2010; e.g., “I often think that a different job would make my life more interesting.”). Thereby, a high level of *commitment* in the workplace is correlated with a stable self-concept, whereas *reconsideration of commitment* is correlated with an unstable self (Crocetti et al., 2008).

In addition to the stability of the self-concept, capturing identity roles is also useful as they permit conclusions about identification possibilities within their work, such as identification with their work group or the organization as a whole (Welbourne, 2012). These are related to satisfaction and organizational behavior (Crocetti et al., 2014) and thus make it possible to derive an individual approach for AI implementation depending on the individual identification profile. Welbourne (2012) defined five roles within organizations that employees can identify with. These include *organizational identity*, which shows a high level of acceptance and identification with organizational values and goals (e.g., “Being proud of the company”). It also includes *workplace identity*, where people identify strongly with their workplace and see their work as an important and central part of their lives (e.g., “Staying in the job that I have now”). Another important identity to consider is *career identity* (e.g., “Doing things that will help me in my career”). This encompasses identification with one's career, which goes beyond identification with one's job and extends across the entire pattern of work-related experiences. In addition, *entrepreneurial identity* is measured, which is characterized by the desire to drive innovation and change, as well as risk-taking behavior (e.g., “Being able to change the ways things are done”). Finally, *team identity* is determined as a strong identification with one's team and a high level of commitment to achieve team-relevant goals (e.g., “Spending time with people in my work group.”). These five work-relevant role identities are surveyed in the JOPI with the help of the Role Based Identity Scale (RBIS; Welbourne, 2012) to further specify the construct of job identity and to derive role-specific interventions for human-centered implementation.

Overall, the second section of the JOPI uses four scales to infer a person's job identity holistically. This is important for a human-centered AI implementation that is aligned with the needs and capabilities of the human part and does not threaten values and activities that a person identifies with within their job. Moreover, if it is possible to identify job-defining activities and preserve them beyond job-specific changes, acceptance and usage will be promoted (Wilkens et al., 2020).

### 3.3. Perception of the workplace

The perception of the workplace is an individual and subjective phenomenon, which can influence the experience, behavior, as well as attitude in the workplace (Stanimir, 2020). How employees perceive their workplace depends on work characteristics and individual preferences (Harter et al., 2010) and their match. The perception of one's workplace is related to wellbeing (Harter et al., 2010; Tausch and Peifer, 2019), motivation (Tausch and Peifer, 2019), job identity (Crocetti et al., 2014), and performance (Yang and Choi, 2014). An organization needs to create a workplace that is perceived positively by employees to promote motivation, wellbeing, and job identity to benefit from associated resources (Wegge et al., 2006). However, the implementation of AI can change work characteristics and activities (Leonardi and Bailey, 2008) and consequently also has an impact on the perception of the workplace (Bhargava et al., 2021). In this context, Bhargava et al. (2021) reported a “satisfaction dilemma” (Bhargava et al., 2021, p. 111) in which employees are satisfied with the benefits of an AI, such as increased fairness or uniqueness of work, whereas they are less satisfied with the impact of AI on work and social life (Bhargava et al., 2021). Which impact predominates depends on the individual's possibility to act according to their job identity and role in the workplace (Bhargava et al., 2021). Hence, it is essential for the transition to human–AI teaming workplaces to capture facets of workplace perception as outcome variables, and not only production key figures. In this way, changes in perception due to the redesign become visible and attention can be drawn to be ensured that the workplace is perceived as conducive to motivation, wellbeing, and identity after AI deployment.

To capture perceptions of a workplace, it is useful to examine job satisfaction because these two constructs reciprocally influence each other (Wong et al., 1998). Job satisfaction describes a positive emotional state that arises from experiences at the workplace and the evaluation of one's workplace (Locke, 1976). One predictor of job satisfaction, in turn, is flow experience (Maeran, 2013). Flow describes an experience of being completely engaged in the fulfillment of an activity (Csíkszentmihályi, 1975). Furthermore, flow is characterized by total absorption, an ideal balance between demands and skills, and enjoyment (Peifer and Engeser, 2021). Another predictor of job satisfaction is occupational self-efficacy (Peng and Mao, 2015), a self-related cognition for assessing one's ability to cope successfully with challenging or difficult situations by one's abilities (Abele and Spurk, 2009) at work. Thus, self-efficacy describes a relatively stable psychological construct that is formed through learning experiences concerning one's abilities (Bandura, 1977). Moreover, work engagement can also be considered a predictor of job satisfaction (Yalabik et al., 2017), which can be defined as an affective-motivational state characterized by dedication and vitality (Schaufeli et al., 2002). Finally, general wellbeing as well correlates with the perception of one's workplace (Hvalič-Touzery et al., 2020). It includes positive feelings such as joy and satisfaction arising from personal and professional experiences (Lee et al., 2007). In addition, there is a correlation between wellbeing and innovation in the workplace (Warr, 2011; Madrid et al., 2014), which makes it an important outcome to predict a person's openness to innovations in the workplace as well. Combining the described constructs may be appropriate for inferring perceptions of one's workplace. Therefore, five scales are surveyed in the JOPI in the section of job perception: *job satisfaction, flow experience, occupational self-efficacy, work engagement*, and *general wellbeing*.

The items of the Job Diagnostic Survey (JDS; Hackman and Oldham, 1974) for general and specific job satisfaction were used as a basis for recording job satisfaction in the JOPI. Similar to the JDS, general and specific facets of job satisfaction are surveyed to be able to form a comprehensive picture of the respondents' satisfaction. We limited collecting facets of specific job satisfaction to those constructs that can be assumed to be influenced by technology implementations. One facet is job security, which often suffers in the context of technologies due to discussions about the replacement of humans by smart technologies (Pollak et al., 2021). Increased use of technology often changes the role of the individual from an active agent to a passive controller (Parker and Grote, 2020; Rieth and Hagemann, 2021), which can result in a loss of capabilities (Frank and Kluge, 2019). Therefore, satisfaction with personal growth and development is also examined. Changing an individual's role in the workplace using technology generally requires different skills from those in the past (Bednar and Welch, 2020). Accordingly, the JOPI measures satisfaction with compensation in relation to the changed requirements and activities.

The constructs of flow, occupational self-efficacy, and work engagement are distinct but closely related constructs (Lisbona et al., 2018). Working conditions that are perceived as conducive to flow, self-efficacy, and engagement can function as work-related resources that lead to greater satisfaction and better quality of work life (Orgambídez et al., 2020), and thus also positively influence general wellbeing (Gupta and Shaheen, 2018). To draw conclusions about motivational support and quality of work life, these four constructs are surveyed in the JOPI with established scales. Flow experience is measured using the Flow Frequency Scale (FFS; e.g.,: “In the last two weeks, how often did you experience joy in your job?”) by Bartzik et al. (2021), occupational self-efficacy using the General Self-Efficacy Short Scale (ASKU; Beierlein et al., 2012; e.g.,: “In difficult situations at work, I can rely on my abilities.”), work engagement using the Utrecht Work Engagement Scale (UWES; Schaufeli et al., 2006; e.g.,: “At my work, I feel bursting with energy.”), and general wellbeing is assessed by using the scale WHO-5 [World Health Organization (2021); e.g.,: “Over the past two weeks I have felt active and vigorous.”].

This third section of the JOPI aims to capture the current state of the motivational and wellbeing supportiveness of the actual workplace. With the help of this information, implementation strategies can be derived. It also allows visualizing the impact of restructured jobs on the perception of affected employees through pre-post comparisons, which provides important clues for optimization and adaptation to human needs.

### 3.4. Evaluation of working with AI

After centering humans with their needs, abilities, and perceptions in this study, the following part deals more intensively with AI technologies and their functionality to describe the last section of the JOPI, which brings the two together. The way an AI is evaluated depends on its functions, interaction capabilities, accuracy, and task appropriateness (Parker and Grote, 2020). Especially in a human–AI teaming workplace, the possibility for interaction and collaboration is crucial, as the workplace design is aimed at collaboration (Kluge et al., 2021). Furthermore, successful collaboration and task distribution are important for the perception of the workplace after the AI introduction (Sadeghian and Hassenzahl, 2022). Thereby, the collaboration, as well as the AI system, is evaluated positively by employees if it supports human capabilities and also takes into account the meaningfulness in terms of job identity (Mirbabaie et al., 2022; Sadeghian and Hassenzahl, 2022). Accordingly, AI should be used in a way that complements human skills (Jarrahi, 2018) and promotes identity (Endacott, 2021; Mirbabaie et al., 2022). This is particularly important in the context of AI technologies, but also more difficult to achieve, as AI differs from other technologies in two main aspects (Endacott, 2021):

- AI technologies “learn” by deriving patterns from large amounts of data.
- These patterns can be applied to different situations over time (Faraj et al., 2018).

In a human–AI teaming workplace, the actions of AI and those of the humans influence each other (Endacott, 2021). This means that the human interacts with the results of the AI's work, or that the AI performs actions on behalf of the human. However, the patterns derived from AI technologies are mostly untransparent to humans, which is why humans must be able to rely on and trust the AI's actions without fully understanding their basis (Endacott, 2021). This can have a negative impact on a person's job identity (Petriglieri, 2011), for example, if the AI represents people in a way that the human would not have chosen. In addition, AI technologies are not able to form multiple identities as humans do, which means that they are not as flexible in adapting their actions to (social) contexts (Endacott, 2021). As a result, the use of AI technologies can limit the dynamics in situations, which might threaten one's job identity. If such a task or context misfit threatens one's identity, the AI is evaluated more negatively and can lead to rejecting behavior (Mirbabaie et al., 2022). Consequently, JOPI should include different facets for the evaluation of the deployed AI, such as collaboration or task–technology fit. In this way, optimization measures can be developed to better adapt AI to the needs, skills, and job identity of the employees, so that AI augments human skills instead of replacing them (Jarrahi, 2018).

To create such an understanding, three self-developed scales are used in this section: *human–AI teaming, task–technology fit*, and *wellbeing in working with the AI system*. They are complemented with one established but adjusted scale to be able to evaluate the introduced AI comprehensively (*evaluation of AI*). These scales, from the section that focuses on the AI used for a task, cover less the technological characteristics of the system, but concentrate more on humans and their perception in the collaboration. This is based on the assumption that successful teaming and an optimal fit between the task and the technology are indispensable (Bevan et al., 2015; Thomaschewski et al., 2020). Of course, technological characteristics and the design of the interface also play a major role in the interaction and perception of the system (e.g., Oulasvirta et al., 2020). However, no matter how well the interface is designed, if there is no fit between the technology and the task, it cannot be compensated for by a good design of the interface (Egbert, 2020). Thus, we identify the variables collected in the last section of the JOPI as requirements for human–technology collaboration that must be given before further adaptions can be made.

The idea of a partnership between humans and technology is that machines should take over everyday tasks so that humans can devote themselves to more creative or identity-giving tasks (Jarrahi, 2018). The degree of fulfillment of this targeted symbiotic collaboration is measured by the human–AI teaming scale. It contains 13 items (see Table 5) that capture the degree to which working with AI is perceived as beneficial to one's work and how employees rate the extent to which AI enables real teamwork.

**Table 5:** Items for measuring human–AI teaming, task–technology fit, and wellbeing in working with the AI.

**No**.	**Item**	**Polarity**	**Scale**
1	When working with the AI system, I feel that the AI system relieves me of tasks that I am less inclined to do myself.	+	HAIT
2	When working with the AI system, we can achieve more together than I or the AI system can alone.	+	HAIT
3	The AI system takes on important complementary tasks that I would otherwise not get to due to lack of time.	+	HAIT
4	When working together with the AI system, we complement each other ideally.	+	HAIT
5	When working with the AI system, everyone can contribute their strengths in the best possible way.	+	HAIT
6	Working with the AI system means that I can concentrate fully on the important aspects of my work.	+	HAIT
7	The AI system takes over tasks that I actually enjoy and would rather do myself.	–	HAIT
8	Working with the AI system leads to me only doing leftover work that actually bores me.	–	HAIT
9	When working with the AI, at the end of the working day I ask myself whether I actually want to continue doing this work.	–	HAIT
10	I feel like a real team when working with the AI.	+	HAIT
11	Working with the AI system allows me to develop more creative solutions that I would not have come up with on my own.	+	HAIT
12	Working with the AI system inspires me.	+	HAIT
13	Working with the AI system makes me feel like I'm part of a real team.	+	HAIT
14	Whoever developed this AI system really thought about how to support my tasks in a meaningful way.	+	TTF
15	It is obvious that someone who understands my work was involved in the development of this AI system.	+	TTF
16	It is obvious that someone who knows my work processes was involved in the development of this AI system.	+	TTF
17	The AI system fits very well with the tasks I do.	+	TTF
18	Working with the AI system is an enrichment for my work.	+	TTF
19	I feel comfortable working with the AI system.	+	WWAI
20	I can imagine that working with such an AI system will be fun for me in the long run.	+	WWAI
21	I can imagine that working with such an AI system will help me in the long run.	+	WWAI

The items were conducted in German. HAIT, human–AI teaming; TTF, task–technology fit; WWAI, wellbeing in working with the AI.

In addition to the possibility of teaming between AI and humans, the system should be designed to support the completion of tasks within the workplace. It is called task–technology fit (Goodhue and Thompson, 1995) and is also surveyed in the JOPI. For a human-centered approach, the technology must be optimally tailored to the task and needs of the employees to optimally support their performance (Trist and Bamforth, 1951; Waterson et al., 2015). To get an indication of how well an implemented technology fits the tasks of employees, the task–technology fit scale was developed (see Table 5).

The third scale in this section addresses wellbeing in the context of working with the implemented AI system. In the treatise on ethically appropriate design of AI systems by IEEE (2019), the end-user's wellbeing is considered as an ultimate criterion in the development of technology to meet the principles of fairness and prevent harm. Therefore, to capture the criterion of wellbeing in collaboration with AI, three items were developed (see Table 5).

Last, this section includes a scale for the general *evaluation of AI*. This scale is important to survey because the evaluation of technology is related to its use and acceptance (Davis, 1989). Knowledge of how people evaluate the AI system can provide a starting point for further improvements of the AI implemented, while a lack of knowledge can lead to AI implementation failing without companies understanding the reasons or being able to readjust. The scale *evaluation of AI* is designed with a total of five items, which were taken from Ajzen and Fishbein (1980) with slight adaptations. The (adapted) iterations of all five items are similar: “I perceive the support by the AI system as...”, rating the AI on a 6-point Likert scale from:

(1) “very bad” to “very good”,
(2) “very foolish” to “very wise”,
(3) “very unfavorable” to “very favorable”,
(4) “very harmful” to “very beneficial”,
(5) “very negative” to “very positive”.

As a result, the last section of the JOPI includes the evaluation of the implemented AI and the cooperation with it. This part of the inventory can only be collected after AI implementation or during its testing phase, as the experience of working with the AI is essential for answering the scales involved. The findings from this section are intended to provide information on the task appropriateness of the AI used and reveal interfaces that could be improved and thus adapted. Due to the small number of scales and items, this section is initially understood as a first screening of the AI. If the screening reveals problems and difficulties regarding the AI used, further methods and investigations should be carried out to identify the key problem. For more information on the development and use of the JOPI with all scales involved, see the Supplementary Table 2.

## 4. Method: measuring the psychometric properties of the JOPI

Our study aimed to develop and evaluate a modular inventory that accompanies a human-centered redesign of workplaces confronted with AI implementation. For this purpose, the composition of the JOPI, the construct validity, and the reliability of the scales it contains was psychometrically evaluated using a real use-case of an AI system.

### 4.1. Procedure of the empirical testing of psychometric properties

For the first validation, we were able to make use of an already-developed AI application that could be evaluated by potential users in an online survey using the JOPI. This application is a program to support speech therapists and their patients in practicing at home and during therapy sessions by offering speaking lessons, evaluating them with artificial intelligence, and collecting data on performance parameters for patients as therapists to work with. Although this case is aimed at a very specific target group, the results of the JOPI can be used and generalized for other jobs and branches after this initial validation.

Thus, we conducted an online study with 197 speech therapists. They were first answering the JOPI item considering their current work situation, to have a baseline measurement of their job characteristics, identity, and perception. Then, they were confronted with a vignette of the existing, ready-to-use AI application that people were then asked to imagine using in their daily work (e.g., “Now imagine being able to use an AI-based speech assistance software in your day-to-day work”; for further descriptions, see Supplementary Table 1). Afterward, we repeated the JOPI measurement to see the changes people were expecting in their work life (e.g., “Please answer all of the following statements under the (imagined) use of the described AI in your everyday work.”). By using this vignette, it was possible to recruit a larger number of participants, regardless of whether they were already working with an AI in their workplace. In addition, it was also possible to test the fourth section of the JOPI for AI evaluation psychometrically and in a standardized manner. Collecting the JOPI using a vignette allowed us to directly capture two measurement points (before and after the AI implementation) without any time delay. This procedure deviates from the intended use of the JOPI insofar as there is actually more time between different measurement points and thus the length of the survey is also substantially reduced. Nevertheless, the use of a vignette allowed us to test all the developed scales psychometrically, which is why this procedure was chosen.

To ensure the quality of the vignette, it was developed with experts, who were already involved in the development of the demonstrated AI application. The vignette consisted of text, image, and video components to maximize immersion (Aguinis and Bradley, 2014) and comprehension (Weber, 1992). In terms of content, the features of the AI application were presented, as well as the resulting potential benefits and what it can be used for.

**Figure 1:**
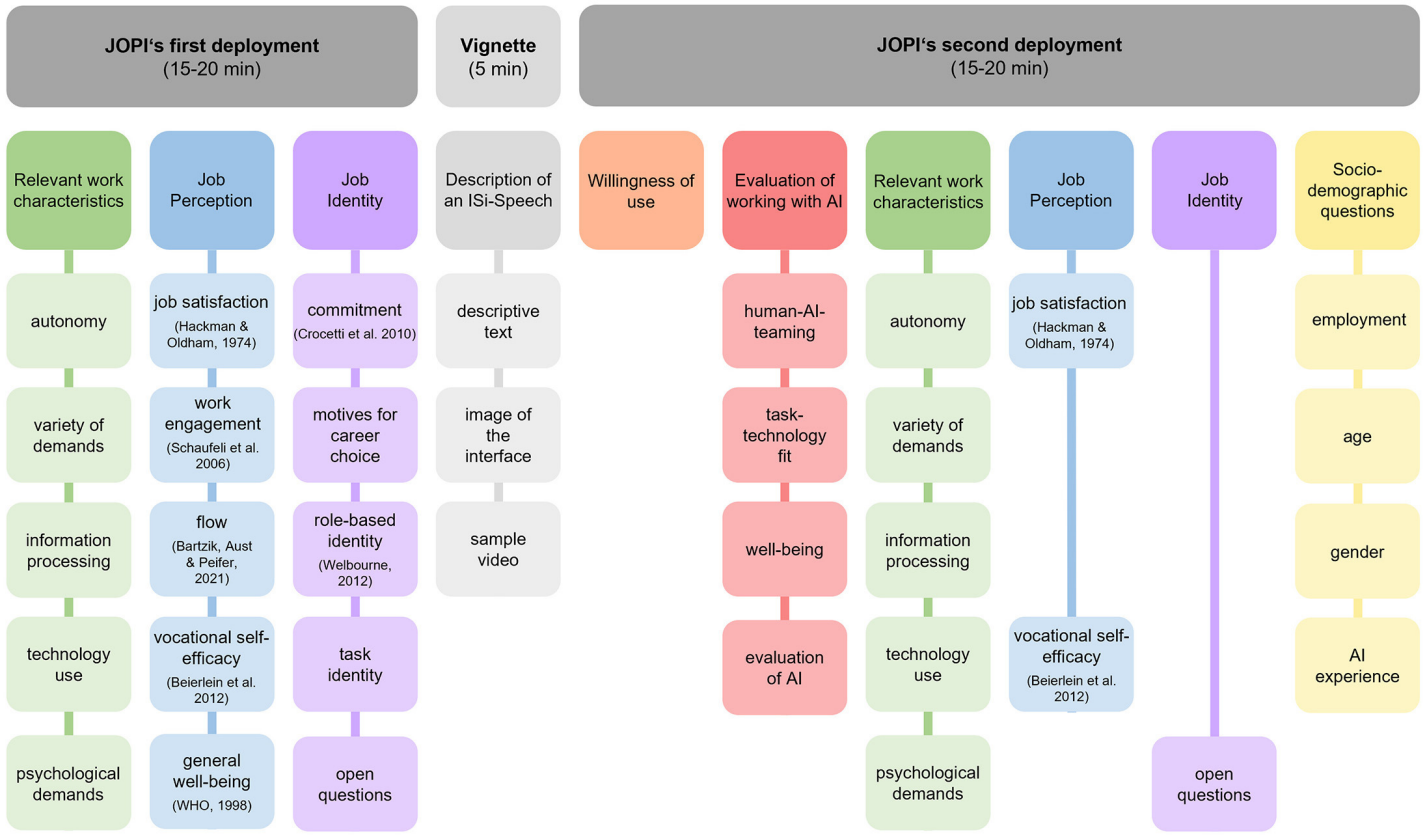
Procedure of the empirical testing of the JOPI.

After informing the participants on the use of the study, they were asked to answer the JOPI scales based on their perception of their current job. This represents the intended first measurement point before the introduction of AI and included the scales from the sections on *relevant work characteristics, job perception*, and *job identity*. In the section on *job identity*, in addition to the described items above, qualitative questions on job identity were asked, in which, for example, the participants were asked to answer what constitutes their role in the workplace. The qualitative questions were included to allow the participants to address aspects of the job identity that were not covered by the quantitative items. The analysis of these will not be discussed further in this study, as it focuses on testing the validity and reliability of the quantitative scales. After this first part of the survey, the participants were confronted with the vignette, in which a real existing AI application was described in detail. Subsequently, the subjects were instructed to answer all the following items under the imagined use of the AI application. The post-vignette survey thus represents the intended second measurement point, after the introduction of AI. First, the willingness to use the presented AI was exploratively surveyed as well as the scales from the section *evaluation of working with AI*. This was followed by a repetition of the scales from the section on *relevant work characteristics* and two scales from the section on *job perception*. The scales were repeatedly collected for direct comparison between the two measurement points. Due to the length of the survey, some scales were omitted from the repeated measurement in this vignette study. In a real application scenario, where there is more time between the repetitions, all scales of the first measurement point should also be collected at the second measurement point. Regarding job identity, only qualitative questions were asked about the extent to which the participants perceived a threat to their identity from the use of AI. The content analysis of these qualitative questions will also not be discussed in the rest of the study. Finally, the participants were asked to answer a few sociodemographic questions concerning age, gender, and AI experience (see Figure 1 for an overview). During the survey, participants were given feedback on how far they had progressed in completing the items to increase motivation to fully complete the survey (e.g., “You have already answered more than half of the questions in this survey!”).

### 4.2. Sample and study design

Data collection took place from 01 March to 05 August 2022. A total of 197 participants were recruited. All of them were speech therapists from Germany, as this is the application context of the AI system used in the vignette. After cleaning the data, which excluded participants with conspicuous response patterns as well as those who had not answered the control questions correctly and those who did not agree with the conditions of participation, a dataset with 156 participants remained. Due to dropouts caused by the length of the survey (*M*_ProcessingTime_ = 85.76 min, *SD*_ProcessingTime_ = 211.86 min, *Mdn*_ProcessingTime_ = 37.83 min, *range*_ProcessingTime_ = 15.52; 1,309.08 min), 66 participants remained who fully completed the survey. Since demographic data were collected at the end of the JOPI, only participants who fully completed the study can be demographically described. Of this group, 62 (93.94%) were female, three (4.54%) were male, and one (1.52%) identified themselves as diverse. This is a typical gender distribution in the job under the survey. The respondents' ages ranged between 21 and 60+ years, determined using predefined categories.

The participants were recruited by e-mail. Participation in the survey was not reimbursed. To qualify for the survey, the participants had to be at least 18 years of age and currently work or have worked in speech-language pathology or related professions, as the AI application described in the vignette was developed for use in speech therapy. The recruited participants had an average professional experience of *M* = 14.62 years (*SD* = 8.46, *range* = 1; 28), of which 21 (31.82%) worked full-time, 12 (18.18%) part-time, 23 (34.85%) were freelancers, eight (12.12%) self-employed, and two (3.03%) were retired; 22 (33.33%) participants had already gathered experience with AI in their daily work and 44 (66.67%) had no experience with AI.

All the participants were informed in advance of the purpose and objectives of the study and gave their informed consent before attending. The survey was approved by the local ethics committee (no. 750) and conducted in accordance with the guidelines of the German Research Foundation (Schönbrodt et al., 2017) for ethics committee confirmation of clearance.

### 4.3. Statistical analysis

Statistical analyses were performed using R and Rstudio (version 4.2.0; RStudio Team, 2022). We conducted an item and scale analysis of all scales involved in the JOPI to test their reliability using Cronbach's alpha. Values between α = 0.7 and α = 0.9 were targeted (Hussy et al., 2010) for sufficiently high internal consistency. We as well-tested for normal distribution using the Kolmogorov–Smirnov test.

For the self-developed scales, the construct validity was examined with the help of an exploratory factor analysis (EFA) using maximum likelihood factor analysis (rotation: oblimin) to draw preliminary conclusions about the quality of these scales. For this purpose, the normal distribution was first tested by analyses of skewness, kurtosis, and the Scree test. Then, by using principal component analysis (PCA), the number of factors was determined, with which the EFA was carried out.

The fit of the scales within the four sections was then calculated to test the composition of the JOPI. To test the consistency of the four sections within JOPI, correlation matrices (spearman-method; significance level set at α = 0.05) were calculated for each section using the scale means. According to Cohen (1988), an effect of *r* = 0.10 is small, *r* = 0.30 is moderate, and *r* = 0.50 is interpreted as a strong correlation.

For this statistical analysis, scale-specific datasets were created with subjects who completed the examined scale entirely. For this purpose, from the total cleansed dataset of *N* = 156, those who fully completed the scale under study were included. That means that the scale analyses were calculated with different numbers of subjects depending on the answers recorded for each scale (*range*_participants_ = 150; 83). This procedure was chosen to increase the robustness of the reliability and validity analyses, as a minimum of 150–200 subjects is recommended for meaningful results (Worthington and Whittaker, 2006).

## 5. Results

Table 6 shows the descriptive statistics of the JOPI and its scales. According to the analyses with the Kolmogorov–Smirnov test, not all scales are normally distributed, although the values of skewness and kurtosis are close to a normal distribution. However, all scales except for the ASKU show a negative skewness. In addition, high mean values (tending toward the upper end of the scale) and low standard deviations can be observed throughout most scales.

**Table 6:** Descriptive statistics of the JOPI.

**Section**	**Scale**	**Range of the scale**	***M***	***SD***	***Sk***	***Ku***	**Kolmogorov-Smirnov**	**α pre adjustment**	**α post adjustment**	***N***
**Work characteristics**										
	Relevant work characteristics	1;6	4.7	0.45	−0.14	−0.51	*p* > 0.05	0.66	0.72	142
**Job identity**										
	U-MICS	1;6	4.55	0.71	−0.39	−0.41	*p* > 0.05	–	0.85	135
	RBIS	1;6	4.38	0.56	−0.26	−0.58	*p* > 0.05	–	0.83	122
	TBIS	1;6	4.26	0.35	−0.32	−0.39	*p* < 0.05	0.73	0.81	120
**Job perception**										
	Job satisfaction	1;6	4.79	0.72	−0.8	0.49	*p* < 0.05	–	0.85	146
	UWES	1;6	4.33	0.92	−0.25	−0.88	*p* < 0.05	–	0.94	137
	FFS	1;6	4.2	0.83	−0.31	−0.75	*p* < 0.05	–	0.92	137
	ASKU	1;6	4.9	0.68	0.08	−1.02	*p* < 0.05	–	0.86	135
	WHO-5	1;6	3.85	0.93	−0.38	−0.41	*p* < 0.05	–	0.84	150
**Evaluation of Work with AI**										
	Human-AI teaming	1;5	3.12	0.81	−0.66	0.19	*p* < 0.05	0.90	0.90	85
	Task-technology fit	1;5	3.54	0.99	−0.74	−0.05	*p* < 0.05	0.91	0.91	84
	Wellbeing working with AI	1;5	3.45	1.07	−0.76	0.05	*p* < 0.05	0.89	0.89	83
	Evaluation of working with AI	1;5	4.27	1.01	−0.71	1.13	*p* < 0.05	–	0.97	90

Sk, skewness; Ku, kurtosis; U-MICS, Utrecht management of identity scale; RBIS, role-based identity scale; TBIS, task-based identity scale; UWES, Utrecht work engagement scale; FFS, flow frequency scale; ASKU, Allgemeine Selbstwirksamkeits Kurzskala (general self-efficacy short scale); WHO-5, World Health Organization—Five Wellbeing Index.

The established scales (U-MICS, RBIS, job satisfaction, UWES, FFS, ASKU, WHO-5, and evaluation of AI) had reliability values of α > 0.80 (Table 6). The following analysis focuses on the self-developed scales (relevant work characteristics, TBIS, human-AI teaming, task-technology fit, and wellbeing working with AI) by estimating values for reliability and checking the factor structure exploratively.

### 5.1. Relevant work characteristics

The scale for recording relevant work characteristics had a reliability value of α = 0.66 after the first analysis with all the items completed. It was found that item 7 (“My work allows me to process incoming information one by one.”) was negatively correlated with the scale even after the pole reversal, which is why this item was excluded from further analyses. After removing item 7, the scale showed an improved internal consistency of α = 0.72. This was followed by testing the number of factors and factor loadings. The Bartlett's test (*X*^2^(55) = 596.90, p < 0.001) and the Kaiser–Meyer–Olkin measure of sampling adequacy (KMO = 0.7) indicated that the variables were suitable for EFA. Using PCA and examining the scree plot, four factors with an eigenvalue of λ > 1 were identified, accounting for 56.7% of the variance (Table 7). Thus, the items loaded on four factors that could be clustered substantively are as follows: *autonomy, cognitive load, psychological demands*, and *technology use*. This clustering largely coincides with the intended subscales. However, it appeared that no factor analytical distinction was made between the variety of demands and information process and that both subscales loaded on one factor. Therefore, these were combined under the factor *cognitive load*.

**Table 7:** Factor loadings of the work characteristics scale.

**Item**	**Factor 1 (cognitive load)**	**Factor 2 (autonomy)**	**Factor 3 (psychological demands)**	**Factor 4 (technology use)**
1		**0.740**		
2		**0.981**		
3	0.185	**0.599**		
4	**0.857**			
5	**0.845**			
6	**0.594**	−0.113	0.183	
7				**0.991**
8	−0.143			**0.449**
9	−0.215	−0.152	**0.549**	
10	0.235	−0.164	**0.411**	
11			**0.997**	

Coefficients assigned to the factor are highlighted.

The internal consistency of the subscales identified here has acceptable values (α > 0.64). Improvements are only needed in the subscale for measuring *technology use* (α = 0.58), that in the end only consists of two items.

### 5.2. Task-based identity scale (TBIS)

The TBIS showed a reliability value of α = 0.73. However, there were also items with negative discriminatory power values after repolarization of the items. This includes items 25–27, which measure activities that are low (cognitively) demanding, and item 16 (for measuring activities that involve hands-on work). Item 16 was, therefore, removed from the item pool. For items 25–27, a correlation matrix was first used to examine the correlation with items 7–9 (for recording activities that involve solving complex problems), as a similarity in content could be assumed (see Supplementary Table 1). It was found that item 27 correlates strongly with items 7–9; therefore, this item was initially retained as a possible extension to this subscale. Items 25 and 26 showed only negligible correlations with this subscale consisting of items 7–9, which is why they were removed from the item pool. After the exclusion of a total of three items (16, 25, 26) due to negative discriminatory power and lack of content fit, internal consistency improved to α = 0.81. The reliability of the assumed subscales showed the values listed in Table 8.

**Table 8:** Reliability of the assumed subscales of the TBIS.

	**CA**	**LG**	**CP**	**HP**	**SC**	**HO**	**JA**	**AR**	**AP**	**AE**
α	0.79	0.62	0.80	0.52	0.44	0.35	0.79	0.54	0.37	0.61

CA, creative activities; LC, activities that promote learning and curiosity; CP, activities that involve solving complex problems; HP, activities that help other people; SC, activities carried out with others; HO, activities that involve hands-on work; JA, activities involving the pursuit of justice; AR, activities involving responsibility; AP, activities that involve high prestige; AE, activities that require a high level of expertise.

Not all assumed subscales showed acceptable reliabilities (HP, SC, HO, AR, and AP). Therefore, the factor structure and factor loadings were exploratorily validated. The Bartlett's test [*X*^2^(435) = 1,365.99, *p* < 0.001] and the Kaiser–Meyer–Olkin Measure of Sampling Adequacy (KMO = 0.66) indicated that the variables were suitable for EFA. Using PCA and examining the scree plot, five factors with an eigenvalue of λ > 1 were identified, accounting for 37.9% of the variance (Table 9). The five identified factors can be clustered in terms of content as follows: *challenging tasks* (from the primal subscales LC and CP), *(pro)social tasks* (from the primal subscales HP, SC, HO, and item 22), *creative tasks* (from the primal subscale CA), *tasks that contribute to society* (from the primal subscales JA and AR), and *tasks related to social status* (from the primal subscales AP and AE).

**Table 9:** Factor loadings of the TBIS.

**Item**	**Factor 1 (challenging tasks)**	**Factor 2 [(pro)social tasks]**	**Factor 3 (creative tasks)**	**Factor 4 (tasks that contribute to society)**	**Factor 5 (tasks related to social status)**
1			**0.805**		
2	−0.156		**0.721**		
3			**0.702**		
4	**0.415**				0.292
5	**0.332**				0.313
6	**0.318**	−0.107	0.328		0.174
7	**0.923**				
8	**0.380**		0.276		0.111
9	**0.596**		−0.123		0.192
10		**0.622**	−0.113	0.146	
11		**0.662**		0.126	
12		**0.165**		0.128	0.153
13		**0.684**	0.125	−0.117	
14		**0.354**	−0.150		
15		**0.296**			
17		**0.622**		−0.120	
18	−0.119	**0.263**	0.237	−0.149	
19				0.779	
20				**0.821**	−0.129
21			0.121	**0.589**	0.111
22	0.184	**0.506**			
23	−0.157	0.172		**0.475**	0.327
24				**0.311**	0.568
27	**0.752**				
28	−0.233		−0.346		**0.419**
29	0.116		−0.255		**0.158**
30		0.173	−0.133	0.121	**0.203**
31	0.161				**0.661**
32	0.127			−0.104	**0.644**
33			0.268		**0.322**
α	0.81	0.70	0.80	0.77	0.56

Coefficients assigned to the factor are highlighted.

The internal consistency of the subscales identified here has good values (α > 0.7; Table 9). However, improvements are needed in the subscale for measuring *tasks related to social status* (α = 0.56).

### 5.3. Human–AI teaming, task–technology fit, and Wellbeing working with AI

Since the self-developed scales from the last section of the JOPI for AI evaluation all showed very satisfactory reliability values (Table 10), no item was excluded. To verify the assumed 1-factor structure, EFA was estimated for each of the scales. The Bartlett's test and the Kaiser–Meyer–Olkin measure of sampling adequacy indicated that the variables were suitable for EFA (Table 10). For the *task–technology fit* and *wellbeing working with AI* scales, all items loaded on one factor with a high variance explanation (Table 10). The *human–AI teaming* scale initially showed two factors. However, closer examination revealed that the inverted items were loaded on the second factor (Table 11). Thus, this factor is only a method factor (DiStefano and Motl, 2006) and has no significance for the content of the scale.

**Table 10:** Scale analysis of the section AI evaluation.

**Scale**	**α**	**Bartlett's test**	**KMO**	**Factors λ > 1**	**R^2^**
Human-AI teaming	0.90	[*X*^2^(66) = 605.70, *p* < 0.001]	0.85	2	0.56
Task-technology fit	0.91	[*X*^2^(10) = 311.47, *p* < 0.001]	0.83	1	0.69
Wellbeing working with AI	0.89	[*X*^2^(3) = 158.62, *p* < 0.001]	0.71	1	0.75

**Table 11:** Factor loadings of the human–AI teaming scale.

**Item**	**Factor 1**	**Factor 2**
1	**0.515**	
2	**0.742**	0.112
3	**0.638**	
4	**0.837**	0.119
5	**0.799**	
6	**0.781**	
7 (–)		**0.649**
8 (–)		**0.994**
9 (–)	**0.285**	**0.606**
10	**0.598**	
11	**0.786**	
12	**0.783**	

Coefficients assigned to the factor are highlighted. Inverted items are marked with a (–).

### 5.4. Composition of the JOPI

After testing the scales within the JOPI, the fit of the scales per section was tested using correlation matrices. Since the first section of the JOPI contains only one scale, the correlations of the scales from Sections 2 to 4 are shown in Table 12. The section scales all correlate moderately or strongly positively with each other.

**Table 12:** Correlations within the JOPI sections.

**Section 2—Job identity**			**Section 3—Job perception**					**Section 4—AI evaluation**		
**Scale**	**TBIS**	**RBIS**	**Scale**	**ASKU**	**FFS**	**UWES**	**WHO-5**	**Scale**	**WWAI**	**TTF**
U-MICS	0.33^**^	0.39^**^	JS	0.57^***^	0.72^***^	0.68^***^	0.68^***^	HAIT	0.85^***^	0.81^***^
RBIS	0.36^**^		WHO-5	0.62^***^	0.75^***^	0.63^***^		TTF	0.79^***^	
			UWES	0.67^***^	0.84^***^					
			FFS	0.75^***^						

Spearman method, significance level set at α = 0.05. Effect of *r* = 0.10 is small (^*^), *r* = 0.30 is moderate (^**^), and *r* = 0.50 (^***^) is a strong correlation. *N* = 66. U-MICS, Utrecht management of identity scale; RBIS, role-based identity scale; TBIS, task-based identity scale; JS, job satisfaction; UWES, Utrecht work engagement scale; FFS, flow frequency scale; ASKU, Allgemeine Selbstwirksamkeits Kurzskala; WHO-5, World Health Organization—Five Wellbeing Index; HAIT, human–AI teaming; TTF, task–technology fit; WWAI, wellbeing working with AI.

In summary, the analyses of the JOPI psychometric properties in that sample show satisfactory results. The assumed factor structures were confirmed to a large extent. Moreover, all scales showed optimal reliabilities after the analyses; only single subscales need further refinements. The sections of the JOPI also demonstrate a coherent picture.

## 6. Discussion

This work aimed to develop an inventory that assesses important aspects of work characteristics, job identity, job perception, and human–technology teaming used to support the implementation of technical systems and especially AI in a human-centered way in a variety of different jobs. The composition of the developed inventory JOPI, as well as the included scales, was psychometrically tested using a vignette of an AI application and its use in everyday work in a speech therapy context. Taken together, the composition of the JOPI and the sections it contains provide reliable results and are useful to survey before and during AI implementation. The self-developed scales also show largely reliable and acceptable psychometric values. In the following, the limitations and boundaries of the work are pointed out. Against the background of the limitations, the results regarding the psychometric parameters of the self-developed scales are discussed. We then transfer our insights to the application in the manufacturing sector and give recommendations for the use of the JOPI there. Further implications are derived and debated.

### 6.1. Limitations of our vignette validation in speech therapy

This study presents the development and first evaluation of the JOPI to provide other researchers as well as practice with an economical, easy-to-use, and scientifically founded instrument. For this, the first step was to check the psychometric values and factor structure of the whole instrument as well as the single scales used. We did this using participants from the same job (speech therapy) and an existing AI system described in a vignette to make the first statements about the quality and applicability of the instrument. Conducting a vignette study has the possibility of low external validity with high internal validity (Aguinis and Bradley, 2014). To increase external validity in vignette designs, a high level of realism is required (Hughes and Huby, 2002), which we reached by using various stimuli (Aiman-Smith et al., 2002) (video and text modules) to present an existing AI-driven app for speech therapists. It also needs an appropriate level of detail (Matza et al., 2021), which we reached by presenting the AI system and its functions in depth in this study. Nevertheless, it cannot be excluded that important details and especially experiences were not covered by the vignette, as an interaction with the AI system was not possible. In addition, it is questionable whether the participants can accurately imagine the changes that the use of technology can entail in the workplace by presenting a vignette. This is especially questionable for participants who have not yet had any experience with the use of AI-supported assistants in their everyday work. This is a problem we have to deal with but that will be addressed differently in further research (see implications). Accordingly, future research should strive to conduct field experiments in which changes caused by an AI application become directly visible and do not remain exclusively based on imagination.

Also critical in this certain application of the JOPI is the length of the vignette study. To be able to implement and validate the intended two measurement points, the use of a vignette was suitable. However, as a result, several scales of the JOPI were repeated directly before and after the vignette, which may have led to response fatigue (Egleston et al., 2011). The resulting length of the survey may explain the high dropout rates (Heerwegh and Loosveldt, 2006) and, in some cases, long completion times. To prevent side effects due to the length of the survey, motivational feedback was given regarding the progress during the survey (Yan et al., 2011). Nevertheless, confounding variables may have interfered with the results due to the length of the survey and possible pauses in the response. The intended use of the JOPI though does not include a vignette as well as no repetition of items in one measurement point, for practical applications, it does not pose a methodological problem.

In addition to limitations in the design of the study, limitations due to the sample collected should also be mentioned. The sample size to conduct the EFA is slightly below the recommended 150 subjects (Worthington and Whittaker, 2006). For the post-vignette scales, the sample size is even below 100 subjects (see Table 6), which also limits the generalizability of the results. Moreover, the psychometric testing of the scales has been conducted in speech therapy only. Thus, the factor structure of the newly developed scales needs to be verified in further studies (as we can see e.g., in the scale on hands-on work) with diverse samples to achieve the goal of developing a cross-industry inventory. In cooperation with further practice partners from the research project this study is a part of, further investigations for more in-depth analyses with more heterogeneous samples are already planned. In this context, refinements of the JOPI's composition as well as extensions are feasible for a comprehensive work analysis tool. Our goal is to have data on the three sections of product-related, people-related, and knowledge-related work with exemplary jobs from each of these sections to map commonalities and differences in relation to AI applications and potentials for human–AI teaming.

### 6.2. Looking at the results

Against the background of the limitations, the findings and their interpretation are discussed in the following. Generally, the compiled JOPI was found to be well-suited for surveying important aspects of technology introductions. There are initial indications of acceptable reliabilities, and testing content validity also revealed good results. The results of these analyses are examined more intensively below with regard to the self-developed scales.

First, the scale for assessing *relevant work characteristics* was psychometrically examined. Exploratory factor analysis identified a four-factor structure consisting of *autonomy, cognitive load, psychological stress*, and *technology use*, matching well with the expected structure. Only the subscales of *information processing* and *variety of demands* showed no differences in the analysis and loaded on one factor that we named *cognitive load*. Cognitive load (CL) describes the way information is processed in working memory and stored in long-term memory in learning or complex situations (Sweller and Chandler, 1991). Three independent sources of cognitive load are distinguished: intrinsic (ICL), extraneous (ECL), and germane (GCL) cognitive load (e.g., Sweller et al., 1998; Brünken et al., 2012). ICL describes the load arising from the complexity of the task to be processed (Sweller, 1994). ECL refers to the load resulting from the design and additional requirements of the task (Klepsch et al., 2017), and GCL is caused by the effort to integrate newly acquired information into existing cognitive schemata (Sweller et al., 1998). Previous research has shown a correlation between the variables *information processing* and *variety of demands* (Okuni and Widyanti, 2019) as well as a correlation with ICL (Klepsch et al., 2017; Okuni and Widyanti, 2019), which supports the results generated in this study and suggests that by combining both variables, ICL can be inferred. Furthermore, a correlation between ECL and *information processing* has also been shown (Okuni and Widyanti, 2019). In the context of technology introductions, the cognitive load may increase due to the change in the status quo of the working memory required to perform the task (Kim and Kankanhalli, 2009; Kumar and Singh, 2016). However, when successfully deployed, the technology can also contribute to a long-term reduction in cognitive load (Baya'a et al., 2018). To be able to estimate the impact, it is useful to survey cognitive load and possible influences of technology implementation in the JOPI. The subscale we developed offers a good starting point, with the help of which ICL and ECL may be deduced.

Overall, the scale for surveying *relevant work characteristics* demonstrated good internal consistency whilst being short and concise, and the identified subscales also showed acceptable values. Only the subscale for assessing *technology use* should be slightly adjusted, especially by adding another item to improve the internal consistency, which is naturally lower with fewer items obviously covering different aspects of a variable.

Work characteristics are important to collect during changes. Depending on their design, these can be perceived as a resource and thus support people in their performance (Peiró et al., 2020). Profound changes in the workplace, such as the introduction of AI or unprepared changes, can influence the design and impact of the work characteristics (Tegtmeier et al., 2022). To contribute to a positive perception, it is useful to investigate the work characteristics and implement AI based on this. This first section of the JOPI provides a good basis for this.

To measure job identity and show potential changes as a consequence of AI implementation, the JOPI contains a number of scales, but the centerpiece is our self-developed scale on task-based identity. Psychometric testing of the TBIS scale revealed a five-factor structure and thus a reduction of the assumed 11 subscales. The subscale measuring low cognitive demanding tasks was excluded from the JOPI because of negative discriminatory power. Causes for this could be inaccurate or insufficient instructions (Moosbrugger and Kelava, 2020). Another explanation might be that the items were not filled in according to the instruction due to social desirability (Moosbrugger and Kelava, 2020) or other response biases that might occur with this specific construct of a preference for “easy” tasks. The task-based identity scale in its revised form consists of five subscales: *challenging tasks, tasks promoting creativity*, (*pro)social tasks, tasks that contribute to society*, and *tasks related to social status*. Two of the original subscales loaded on the first factor: The items capturing tasks that promote curiosity and learning in the workplace and the items capturing tasks that involve solving complex problems. The literature also shows a proximity between the two constructs of curiosity and complex problem-solving (Litman and Jimerson, 2004; Powell et al., 2016), with curiosity discussed as a catalyst for engagement with complex problems and challenging tasks (Horstmeyer, 2019). Thus, combining these original subscales into one factor as *challenging tasks* is consistent with findings in the literature. Tasks promoting creativity remain, as expected and constructed in the original questionnaire, a subscale on its own, being distinct from the others. The subscale tasks involve helping others, interacting with others, and working in a practical manner, as well as one item from the scale that records tasks involving taking responsibility, combined with the new subscale of (pro)social tasks. This clustering of scales is consistent with previous research insofar as interacting with others is a prerequisite for helping others (Sozinova et al., 2017). Furthermore, taking responsibility is also closely related to helping behavior (Zuckerman et al., 1977; Greitemeyer et al., 2006). These three correlating constructs of interaction, helping others, and taking responsibility are united by the construct of *prosocial behavior*, which can therefore be seen here as the superordinate factor (Silva et al., 2009). Merely the items for the recording hands-on work do not fit well with the other items of this factor. This can be explained against the background of the logopaedic sample with the help of which the scales were validated and factor-analytically checked. For speech therapists and related professions, speech therapy is a form of practical work (Franz et al., 2020), which is closely related to interaction and helping others. This explains the composition of this factor and is also understandable against the background of the sample, but may differ in other occupations, e.g., the manufacturing industry. That is why, for further investigations, we will test the hands-on work items again as a separate subscale, sticking to our initial differentiation.

Another identity-relevant task group, as to our analyses, is that of *tasks contributing to society*. We find that items on tasks involving the assumption of responsibility and on tasks involving the pursuit of justice belong to this task group. The literature also shows a close relationship between taking responsibility and exercising justice (Schmitt et al., 2005, 2010), matching our conventionalization. The last identity-relevant task category is that of *tasks relevant to social status*, including the items on tasks that involved high prestige and expertise. According to the dominance-prestige concept, a distinction is made between two ways of achieving high social status: (1) first, with dominance, in which violence or intimidation is used to create fear, or (2) second, with prestige, through sharing expertise and knowledge (Cheng and Tracy, 2014). Based on this approach, the two concepts of prestige and expertise are closely related and mutually dependent, which goes in line with the results of this work. Taken together, the TBIS for capturing tasks people identify within the workplace could be reduced from the original 10 subscales to five. The clustering of the factors is consistent with findings from previous research works. Only the assignment of the subscale for recording practical tasks in the work context as well as for surveying tasks for the assumption of responsibility requires closer examination. In addition to a reasonable factor structure, the developed scale also demonstrated acceptable reliability values. Improvements regarding internal consistency are only necessary for the last factor presented for recording *tasks in relation to social status*. Those will be done in upcoming research.

The scales that we used to measure *human–AI teaming, task–technology fit*, and *wellbeing working with AI*, all proved to be consistent in themselves, except for a method factor that can be ignored on a content level when applying the scale. Thus, all three scales showed very good internal consistency, do not require major revision, and can be used to capture the first impression of an evaluation of the AI by workplace holders.

**Figure 2:**
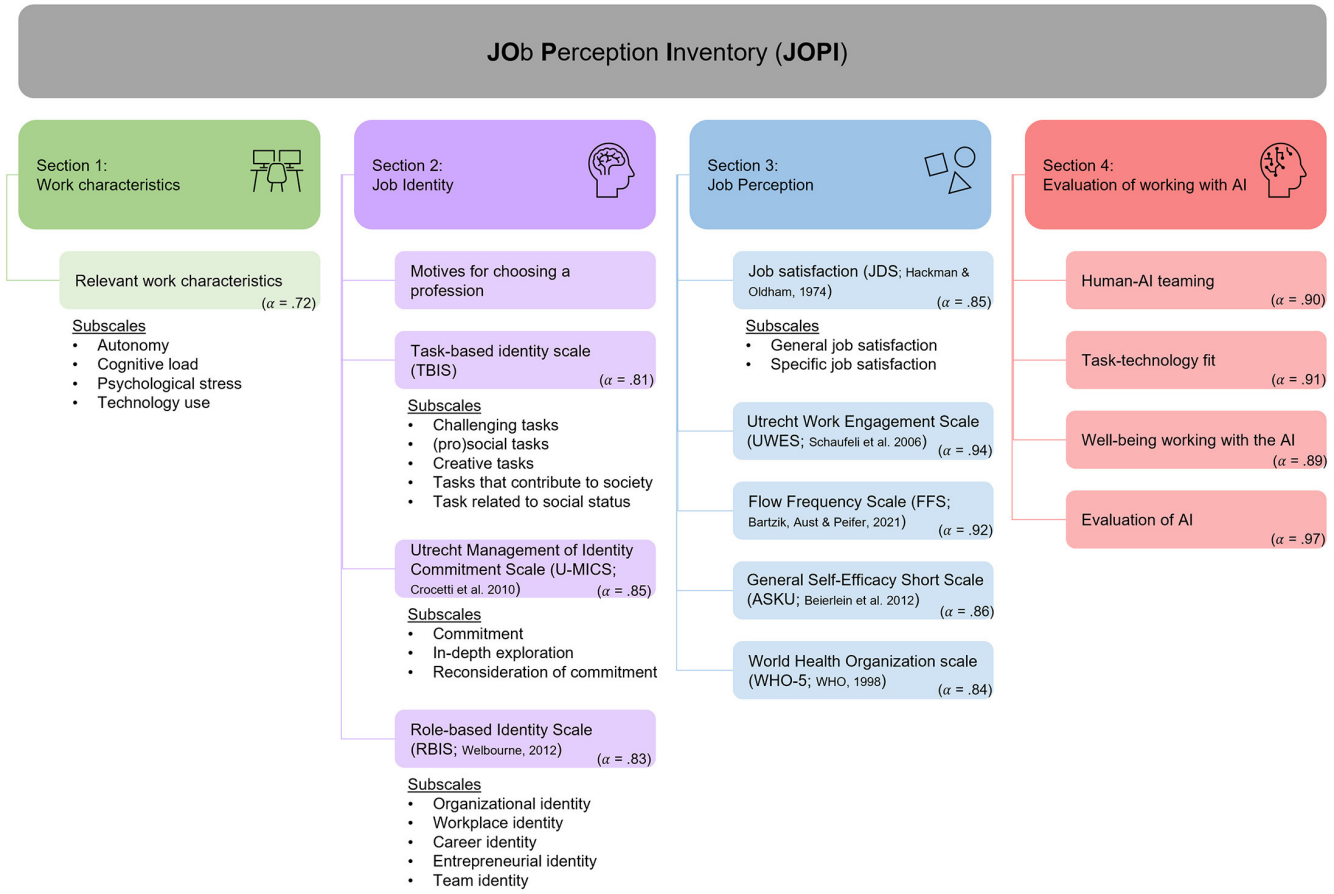
Compilation of the JOPI post adjustment.

After this initial testing of the compilation of the JOPI, a coherent survey inventory emerges which, as expected, captures four sections of technology adoptions. The sections revealed a coherent structure and also the self-developed scales which demonstrated mostly the intended subscales. The scales from the section for the *evaluation of working with AI* all showed one subscale as assumed. The scale for the survey of *relevant work characteristics* revealed four subscales instead of the expected five. The largest deviation was shown by the TBIS scale, in which only five of the 11 assumed subscales were found in this analysis. The changed composition is shown in Figure 2.

### 6.3. JOPI in manufacturing

One important application scenario for the JOPI is manufacturing work, where the implementation of AI systems can, e.g., be used to optimize resource usage, plan workflows, or adapt machine settings to changes in inputs (Wuest et al., 2016; Wang et al., 2018; Aggour et al., 2019). Especially in smaller-scale manufacturing with quite some manual tasks, AI offers the opportunity to reduce distribution costs and increase flexibility, and thus can also change human work, for better or for worse (Jones and Henderson, 2022). In this section, we want to explain the potential use and importance of the JOPI in manufacturing as well as the differences in comparison to our validation use case and sample. For this, we use an example from production which is created by us to illustrate JOPI use and its effects.

Imagine working in a company that produces switch boxes for industrial customers. There is a planning team of engineers who develop the circuit diagrams based on the customer's requirements. These paper-based plans are given to manufacturing workers, who use them to assemble the required materials, mount components, install them in the switch box, and wire them. Each worker follows their personal assembly strategy and incorporates individual experience. In the end, each switch box is quality-checked. Within this process, the company now introduced an AI system to support the interface between the engineers' circuit diagrams and the assembly process. The system can automatically extract an optimal assembly procedure for each digital circuit diagram. Henceforth, the diagrams are displayed on tablets and the AI system provides step-by-step instructions and recommendations for the most resource-efficient assembly procedure. It also makes predictions for subsequent maintenance. This should optimize production times, eliminate ambiguities, and reduce complexity.

In this example, we see great potential for the use of AI from a business point of view, but also great potential for improving work and keeping it motivating and attractive for employees. Nevertheless, the last point is only true if the implementation is intentionally designed with human needs and the human workplace in mind and not if the focus lies on the technology. Therefore, our recommendation would be to use the JOPI as a validated tool for designing human–AI workplaces. Table 13 outlines potential positive and negative changes in the workplace due to AI deployment in our example based on the JOPI sections.

**Table 13:** Possible changes in the workplace in manufacturing due to AI implementation.

**Section of the JOPI**	**Potential positive changes**	**Potential negative changes**
Work characteristics	Technology use would increase, which could result in new skills (Roth et al., 2017) when workers are understanding the principles and decisions of the AI and potentially how to teach it.	AI takes over the decision in which sequence and with which tools the switchgear is produced, which could reduce the workers' perceived autonomy (Welfare et al., 2019). The cognitive load may also decrease (Nazareno and Schiff, 2021), as only the AI instructions have to be followed—resulting in boring, unchallenging work.
Job identity	Hands-on activities, which might be a satisfying and identity-establishing component of the work, can still be pursued. The amount of practical work could even increase (Reiman et al., 2021) because less time has to be invested in planning the switch box construction, which is taken over by the AI.	Problem solving could become irrelevant or even impossible (Wichtl et al., 2019), as the precise AI instructions provide less opportunities for it. If workers see this as the core of their job, their identity might be threatened.
Job Perception	Satisfaction with equipment in the workplace could increase using innovative technology, such as AI. Flow experience could rise due to step-by-step tasks with clear goals (Oliveira et al., 2021).	Satisfaction at work and especially with prospects could decline as AI takes over a large part of the work, making workers feel easily replaceable and less relevant (Bauer and Klapper, 2019).
Evaluation of Work with AI	If the AI perfectly fits the tasks to be executed and is seen as a useful support, human-AI teaming can evolve (Bao et al., 2021).	If the work between humans and AI is perceived as “side-by-side” instead of “together”, workers' wellbeing and performance may suffer (Kemény et al., 2021).

The example of the manufacturing industry shows that the introduction of AI is affecting the workers and their perception of work by all means. If its introduction is based solely on logical considerations, using pre-defined AI solutions and not a systematic implementation process involving the affected employees tends to result in a negative development of the workplace. The resulting side effects can be manifested in reduced wellbeing as well as reduced performance (Reiman et al., 2021). To prevent such effects in the manufacturing industry, it is necessary to deeply understand the existing work system and the emerging socio-technical system, taking into account both the technical and the human perspectives (Sony and Naik, 2020). By doing so, we can unlock all the potential positive changes shown and improve workplaces. For the consideration of the human part, the JOPI as a tool for collecting relevant aspects regarding technology introductions is suitable to implement AI in the manufacturing industry in a human-centered way. Due to the satisfactory reliability and validity values of the JOPI scales, we can assume that their use in manufacturing will also provide helpful results. However, specific conclusions in terms of content are presumptions that need to be explored in more depth in subsequent work.

#### 6.3.1. Implementing AI in the manufacturing industry using the JOPI

For those wanting to use the JOPI in manufacturing, we recommend using it first before the introduction of AI, ideally at that time, when the decision is made to integrate some sort of AI in the workplace. This first version of the JOPI should contain sections for relevant work characteristics, job identity, and job perception, framed in a way that the workers will understand why they are asked about their opinion on their current jobs. It is important to understand how the sometimes abstract questions relate to their work and the changes implemented by an AI, especially for those people not used to surveys. In this way, interesting insights can be gained about workplace resources and stressors as well as the identity-establishing parts of work. If these insights regarding preferences and identifications are captured early in the implementation process, they can (and should) already be considered in AI development or adaptation. For instance, in our switch box example, an interface for reporting and describing problems could have been integrated right from the start, and step-by-step instructions could have been included as an optional function to align AI functions with the workers' needs of autonomy in planning and contributing their experiences. With the consideration of job identity and a human-friendly design of the work characteristics, a decrease in job satisfaction, as well as engagement or commitment, is not to be expected. Furthermore, it can be assumed that providing opportunities to communicate with the system and getting involved can lead to the feeling of true collaboration. The early involvement of employees using JOPI allows for faster implementation of changes in the workplace, as the needs and capabilities of the individual are considered from the very beginning. This avoids multi-step evaluation loops of AI, allowing organizations to return to their daily operations without harming performance and humans with their needs, capabilities, and motivations. After the AI has been deployed, further surveys involving the JOPI should be conducted to continuously improve collaboration and to best adapt the system to the needs and skills of the individual.

Combined, our final recommendations for implementing AI into manufacturing workplaces are as follows:

1. Analyze the existing work detecting resources and stressors within the work characteristics.
2. Understand your workers' job identity.
3. Use those insights to select, design, or configure an AI that will help your workers with their daily tasks in a way that is both efficient and helpful to them.
4. Protect and support their work identity by sustaining identity-relevant tasks and supporting or replacing non-relevant tasks with the use of AI.
5. Analyze work characteristics and job perception after AI implementation again to identify optimization potentials and take appropriate action.

These recommendations can be realized with the use of the JOPI. The goal is to expand and specify these recommendations through future work in the field to provide best practice recommendations for AI implementation in the manufacturing industry and beyond.

### 6.4. Implications

This work highlights the importance of human-centeredness in the workplace when implementing technology and presents an inventory to help to address this issue. While technology implementations in the manufacturing industry are largely technology-centric (Kopp et al., 2016), human-centered approaches are becoming increasingly important in the context of AI technologies. Due to the human-like capabilities of smart technologies (e.g., Huang et al., 2019; Rai et al., 2019; Dellermann et al., 2021), the introduction of those leads to growing complexity in the workplace (Huchler, 2022). For example, the implementation of an intelligent robot can enable direct interactions with humans in the workplace, which in turn allows a flexible allocation of tasks (Tausch et al., 2020). Such an adaptive task allocation brings enormous benefits for the human and the quality of the work (Tausch et al., 2020), and also increases the complexity in the workplace. Given the increasing complexity and possibilities of usage, it is necessary to address the identity-forming and motivation-promoting factors of a workplace while implementing intelligent technologies to meet the needs and capabilities of the employees, and thus to be able to benefit from the synergies of cooperation (Kluge et al., 2021). For this purpose, the present work contributes by deducing an inventory from existing findings of psychological work research, which should accompany human-centered implementation in the workplace. This is relevant in the manufacturing sector against the background of the announced Industry 5.0 (European Economic and Social Committee, 2021). The introduction of a new era, Industry 5.0, is based on the observations that Industry 4.0 is technology-driven and focuses on increasing the efficiency and flexibility of production (Xu et al., 2021). Industry 5.0 is therefore intended to change the technology-driven approach to a value-driven concept (Breque et al., 2021) in which humans and machines work together in a symbiotic relationship (Longo et al., 2020) and which focuses on the human being in the design and collaboration (Bednar and Welch, 2020).

Implications for research arise for further refinements of the JOPI. A few subscales require more items after psychometric testing to be measured reliably, such as the subscale *technology use*. Other scales need further research concerning the resulting factor structure, especially the TBIS scale. Furthermore, it would be useful in future research to apply the JOPI in different occupational groups and to use the results besides the adjustment of the JOPI for the derivation of occupation-specific identity profiles. With the help of these profiles, occupational group-specific recommendations for the implementation of AI could be developed. More in-depth analyses complementing the JOPI would also be useful. Although the sections of the JOPI are already surveyed by several scales, it is still worthwhile to conduct more detailed analyses to identify negative impacts concerning AI introductions and deal with them appropriately. Therefore, in addition to the self-reporting method used in the JOPI, it would be helpful to develop other methods that enable a different approach to the impacts of AI implementations, such as work observations or interviews with external stakeholders. These methods can be combined with the JOPI to form a toolbox consisting of varying methods of psychological work analysis. The JOPI functions as a broad screening of the work context, based on which further in-depth analysis methods can be selected for more detailed elaboration.

## 7. Conclusion

This work contributes to the compilation of an inventory that enables the derivation of human-centered implementation strategies for AI systems at work as well as the work analytical accompaniment of such implementations. The JOPI is thus a tool of psychological work analysis based on self-report. Due to the multiple measurement points, the JOPI does not only provide a selective result on the status quo but also a starting point for a continuous evaluation of the workplace. In this way, the JOPI supports companies in the development of human-centered workplaces with AI, which is necessary for consideration of industry 5.0 and the growing complexity due to intelligent technologies. However, further research is needed to survey the JOPI in more diverse occupational groups and see if consistent results emerge. Additional data can also be used to derive refinements for the composition as well as to develop further methods of job analysis in addition to the JOPI. The aim of future work should be to bundle these different methods into a toolbox that includes various methods of analysis that contribute to human-centered technology implementations.

## Data availability statement

The raw data supporting the conclusions of this article will be made available by the authors, without undue reservation.

## Ethics statement

The studies involving human participants were reviewed and approved by Local Ethics Committee of the Faculty of Psychology, Ruhr University Bochum. The patients/participants provided their written informed consent to participate in this study.

## Author contributions

SB and AT were in charge of data collection and analysis of the study. SB took the lead in writing the paper. AT and AK also wrote parts of the paper and revised the draft several times up to the current state. CP read the content of the paper on several occasions, critically reviewed it, and adjusted it with respect to important intellectual content. All authors participated in the conceptual design of the study and contributed essential parts, such as questionnaire scales and in the written elaboration and made important contributions to the presented result. All authors contributed to the article and approved the submitted version.
