# Supplementary material for: The Job Perception Inventory: considering human factors and needs in the design of human–AI work
Source: Front Psychol. 2023 May 23;14:1128945. doi: 10.3389/fpsyg.2023.1128945 (PMC10243195; doi:10.3389/fpsyg.2023.1128945)
Supplement: Supplementary file 1 [file Table_1.DOCX]

Supplementary Material

The Job Perception Inventory: Considering Human Factors and needs in the design of human AI work in manufacturing industry

Sophie Berretta*, Alina Tausch, Corinna Peifer, Annette Kluge

*** Correspondence:** Sophie Berretta: sophie.berretta@ruhr-uni-bochum.de

# Supplementary Data: Formulation of the vignette for the AI application

Having asked you about your current work situation and what your job entails, we are now interested in possible future changes in your job profile and how you assess them. One major development affecting many professions is digitization. It is also playing an increasing role in speech therapy. In this survey, we are particularly interested in the use of intelligent software systems (so-called AI), which can be used as support in everyday work, for example in the form of apps for speech training.

In the following, we therefore present an app that is currently under development and can support the work of speech therapists in the outpatient care of patients. We are interested in your opinion about the app.

Now imagine that you can use an AI-based speech assistance software in your daily work for patients. This software is called ISi-Speech. ISi-Speech can be individualized and is intended to be used by patients in rehabilitation for people with speech intelligibility impairments and as a supplement to therapy services. This can relieve you as a speech therapist and provide additional information about home exercises and therapy success. ISi-Speech makes it possible to adapt individually to the abilities of the respective patients and to provide them with exercises for the therapy of articulation, prosody, speech volume, speech rate, voice volume and respiration based on this. The training program uses automated speech recognition to provide patients with feedback on the intelligibility of their speech during the exercises.

Patients benefit by being able to train their speech performance independent of time and location. In addition, they can receive objective feedback on their speech performance independently of the therapist.

As a speech therapist, you benefit from ISi-Speech in that you can influence the quality and quantity of the results by having access to the individual patient's results. Already during the training in the therapy situation, results become objectively comparable and can support the follow-up diagnostics. The results of the patient's own training become comprehensible through shared access to the exercise sequences.^[[1]](#footnote-1)^

# Supplementary Figures and Tables

**Table 1**

*Correlation matrix between items 7-9 and items 25-27 of the TBIS*

|  | Task-based identity | Item 07 | Item 08 | Item 09 | Item 25 | Item 26 |
| --- | --- | --- | --- | --- | --- | --- |
| Item 07 | solving complex problems | 1 |  |  |  |  |
| Item 08 | solving complex problems | .48 | 1 |  |  |  |
| Item 09 | solving complex problems | .50 | .37 | 1 |  |  |
| Item 25 | low (cognitively) demanding | -.05 | -.02 | -.16 | 1 |  |
| Item 26 | low (cognitively) demanding | -.26 | -.28 | -.09 | .15 | 1 |
| Item 27 | low (cognitively) demanding | -.77 | -.41 | -.49 | .12 | .18 |

1. The vignette was originally raised in German. The text shown here is a direct translation. [↑](#footnote-ref-1)
