# Supplementary material for: The Job Perception Inventory: considering human factors and needs in the design of human–AI work
Source: Front Psychol. 2023 May 23;14:1128945. doi: 10.3389/fpsyg.2023.1128945 (PMC10243195; doi:10.3389/fpsyg.2023.1128945)
Supplement: Supplementary file 2 [file Table_2.DOCX]

Supplementary Report

The Job Perception Inventory: Scales and Usage

Sophie Berretta*, Alina Tausch, Corinna Peifer, Annette Kluge

*** Correspondence:** Sophie Berretta: sophie.berretta@ruhr-uni-bochum.de

*Note*. For details about the scale development see:
Berretta et al., (under review). The Job Perception Inventory: Considering Human Factors and Human Needs in the design of human AI work. *Front. Comput. Sci.*

*Terms of Use*. The survey inventory is a research instrument that serves research, teaching and practice. It is made available online and protected by copyright, i.e., the copyright remains with the author(s). In addition, please note that the survey inventory makes partial use of previously published scales by other authors. The respective scales are marked below, and the corresponding references can be found at the end of the document.

**Table of contents**

1 Background 3

2 Concept of the JOPI and Usage 4

2.1 Structure of the inventory 4

2.2 Application 5

2.3 Target Group 5

2.4 Requirements 5

2.5 Evaluation procedure 5

2.6 Evaluation aids 6

2.7 Items 6

3 Test construction 14

3.1 Development of the JOPI 14

3.2 Study sample 14

4 Quality criteria 16

4.1 Objectivity 16

4.2 Reliability 16

5 Conclusion 17

References 18

# Background

The adoption of technologies, and in particular intelligent technologies, including Artificial Intelligence (AI), is expected to lead to tremendous organizational benefits (Brynjolfsson & McAfee, 2014). However, technologies are often implemented into the workplace without addressing the needs, skills, and job identity of employees (Kopp et al., 2016). Past experience has shown that such a technology-centric approach can result in various unintended side effects on the human side, as loss of attention (Parasuraman et al., 1993), reduced competencies among employees (Frank & Kluge, 2019) or diminished well-being and motivation (Tausch & Peifer, 2018). These can be counteracted by a human-centered AI development and implementation in the sense of a human-AI teaming approach, which enables a real collaboration between humans and AI (Kluge et al., 2021). This teaming approach focuses on the needs and capabilities of employees, and the workplace and the technologies used should be favorably aligned with these aspects (Huchler, 2022; Jarrahi, 2018). In this regard, the technology implemented is seen as augmentation to the individual in order to support them in their identity and motivation-promoting activity fulfillment (Jarrahi, 2018). Also from an organizational perspective, it is beneficial to consider the human part when introducing and using new technologies, as employees who feel comfortable in their workplace and who are optimally stimulated contribute to positive outcomes (Yang & Choi, 2014).

Although the idea of collaboration between humans and AI has proved useful, its realization is so far insufficiently researched. For this purpose, the Job Perception Inventory (JOPI) as a tool of human-friendly AI implementation is developed. Berretta’s et al., (2023) publication, on which this report is based, describes this theoretical derivation in more detail, as well as the development and validation of the included scales of the JOPI. The full questionnaire instrument as well as additional information on the application of the JOPI are provided in this supplemental report.

# Concept of the JOPI and Usage

## Structure of the inventory

The Job Perception Inventory considers four sections: 1) Work characteristics, 2) Job identity, 3) Perception of the workplace, and 4) Evaluation of working with AI. Each section contains several scales that can be modified in a modular way to suit the context of application. Some of the scales are our own development, whilst other are established, non-commercial scales that are applicable to an AI implementation scenario. The inventory, if fully used, includes 146 items (number of items in parentheses):

1. Relevant work characteristics (k = 11)
   1. Autonomy (k = 3)
   2. Cognitive load (k = 3)
   3. Technology use (k = 2)
   4. Psychological demands (k = 3)
2. Job identity (k = 75)
   1. Motives for choosing a profession (k = 12)
   2. Task-based identity scale (k = 30)
   3. Utrecht Management Of Identity Scale (Crocetti et al., 2010; k = 13)
   4. Role-based Identity Scale (Welbourne, 2012; k = 20)
3. Perception of the workplace (k = 34)
   1. General and specific job satisfaction (Hackman & Oldham, 1974; k = 8)
   2. Flow Frequency Scale (Bartzik et al., 2021; k = 9)
   3. General Self-Efficacy Short Scale (Beierlein et al., 2012; k = 3)
   4. Utrecht Work Engagement Scale (Schaufeli et al., 2006; k = 9)
   5. General well-being (World Health Organization [WHO], 2021; k = 5)
4. Evaluation of working with AI (k = 26)
   1. Human-AI teaming (k = 13)
   2. Task-technology fit (k = 5)
   3. Well-being in working with the AI system (k = 3)
   4. Evaluation of AI (k = 5)

Whilst only the full application with all scales and items can guarantee avoiding a one-sided perspective in AI implementation, modulations can be made, especially with iterations of the JOPI.

## Application

Application of the JOPI is recommended when a work redesign towards a human-AI teaming workplace is required or intended.

The first deployment should ideally take place prior to the introduction of the AI system in order to derive workplace-specific criteria for the motivation- and identity-enhancing design of AI. The JOPI thus defines the target state after the introduction of AI or areas to improve the current work situation. Subsequently, the inventory should be used again during and after AI implementation in order to evaluate the redesigned workplace and if necessary to make further modifications.

The single application of the JOPI with all scales listed takes approximately 20 minutes. Please note that the items were originally formulated and validated by us in German and translated versions are presented (except for the scales that were originally English language).

The items in form of statements can be presented to the participants as a paper-pencil questionnaire or online.

## Target Group

Target group are employees who are concerned by the AI deployment and reorganization at the workplace. In this regard, the JOPI is not limited to a specific application context but can be used in various industries.

## Requirements

The use of the JOPI does not require any special prerequisites or qualifications, except for general experience with questionnaire application and evaluation. It needs a person who adapts the composition of the JOPI to the current situation, who disseminates the survey and evaluates the results. Therefore, a degree of familiarity with actual circumstances should exist, so that modifications can be applied, if necessary. Knowledge of inferential statistics is, as well as an understanding of the jobs at hand and a general understanding of the AI applied, useful for the evaluation and interpretation of results.

## Evaluation procedure

For the analysis, the data preparation includes reversing the polarity of the negatively poled items. Then, mean scale values are calculated for the individual subscales and their interpretation is carried out separately and as a synopsis. The evaluation of the scale values is based on the content level. For example, in the case of the scale for work characteristics, a high score for autonomy is desirable, whereas a high score for psychological demands is evaluated as negative.

## Evaluation aids

No special evaluation aids are available. In the case of large samples, it is recommended to use statistical software to calculate the dimension values (e.g., R and RStudio or SPSS) for time-saving reasons. A manual evaluation is also possible.

For the evaluation and interpretation of multiple JOPI measurements (e.g., before and after an AI implementation), we recommend a graphical analysis of the resulting parameters in order to visualize changes at a glance. Thereby, the focus of the evaluation should primarily be on the changes in the measured values, i.e., on possible increases or decreases. Such changes in the values indicate changes in the perception or the circumstances due to the AI deployment, which should be addressed in further restructuring measures.

## Items

1. Relevant work characteristics

Instruction: This section is about the description of your work tasks. For each statement, please mark the extent to which it applies to you personally.

Response Scale: 6-point Likert scale from “strongly disagree” to “strongly agree”.

**Table 1**

*Items to assess relevant work characteristics*

| **No.** | **Item** | **Polarity** | **Scale** |
| --- | --- | --- | --- |
| 1 | At work, I have a lot of freedom in terms of planning and performing my tasks. | + | AT |
| 2 | I can decide a lot of things independently at work. | + | AT |
| 3 | The way I manage my work tasks is up to me. | + | AT |
| 4 | My work allows me to bring a variety of different skills to the table. | + | CL |
| 5 | I do a lot of different tasks at work that challenge me in different ways. | + | CL |
| 6 | I have to simultaneously look at a lot of information in my work. | + | CL |
| 7 | Part of my job is to use technologies. | + | TU |
| 8 | In my work, I do not rely on the use of technology. | - | TU |
| 9 | I work under high time pressure. | + | PD |
| 10 | My work is emotionally demanding. | + | PD |
| 11 | In my job, I have to manage a large workload. | + | PD |

*Note*. AT = autonomy. CL = cognitive load. TU = technology use. PD = psychological demands.

1. Job identity

*Motives for choosing a profession*

Instruction: Which of the following factors were important to you when choosing a profession? Order the possible reasons in order of importance. The most important point for your career choice should be at the top and the least important point at the bottom.

**Table 2**

*Motives for choosing a profession*

| **No.** | **Item** |
| --- | --- |
| 1 | Opportunity to earn a high salary |
| 2 | Number of hours worked per week |
| 3 | Opportunity for career advancement |
| 4 | Working with people |
| 5 | Helping people |
| 6 | Variety at work |
| 7 | Opportunity to take on responsibility |
| 8 | Self-realization |
| 9 | Interest in work content |
| 10 | Expectation of others |
| 11 | (Family) tradition |
| 12 | Compatibility with family |

*Task-based identity scale (TBIS)*

Instruction: Below, we would like you to identify particularly important characteristics of your job. Please evaluate the statements as honestly and openly as possible.

Response scale: 6-point Likert scale from “strongly disagree” to “strongly agree”.

**Table 3**

*Items for measuring task identity*

| **No.** | **Item** | **Polarity** | **Scale** |
| --- | --- | --- | --- |
| 1 | Being creative is very important to me in my professional life. | + | CA |
| 2 | If possible, I try to avoid creative tasks in my professional life. | - | CA |
| 3 | When I decided on my current profession, creativity played a big role. | + | CA |
| 4 | Learning something new excites me. | + | CT |
| 5 | I am not very interested in acquiring new job-related knowledge. | - | CT |
| 6 | One of the main reasons for choosing my current job is that my curiosity is encouraged there. | + | CT |
| 7 | I like to solve very complex problems. | + | CT |
| 8 | In my everyday work, I prefer tasks that can be solved without much thinking. | - | CT |
| 9 | I chose my current job primarily to deal with complex problems. | + | CT |
| 10 | I like to solve complicated and thought-provoking tasks. | - | CT |
| 11 | Supporting others through my work is very important to me in my professional life. | + | PT |
| 12 | I consciously chose my current job to be able to help others. | + | PT |
| 13 | Helping others does not play a big role in my professional life. | - | PT |
| 14 | The most important thing for me in my professional life is to interact with other people. | + | PT |
| 15 | I chose my current job because of the social contacts. | + | PT |
| 16 | I prefer working alone. | - | PT |
| 17 | I chose my current job because it allows me to work practically. | + | PT |
| 18 | It is important to me to be able to "lend a hand" in my profession. | + | PT |
| 19 | I want to use my work to actively fight injustice. | + | PT |
| 20 | It is important to me to promote the well-being or safety of others through my work. | + | PT |
| 21 | I chose my current job to be able to reduce injustice. | + | CP |
| 22 | I don't think much about the extent to which my work affects the (un)fair treatment of people. | - | CP |
| 23 | I chose my current job to be able to contribute to society. | + | CP |
| 24 | The social contribution of my work is not important to me. | - | CP |
| 25 | I like being admired by others for my work. | + | SC |
| 26 | I chose my current job because it comes with a high reputation. | + | SC |
| 27 | I don't care what others think of my work. | - | SC |
| 28 | It is important to me to use my competences in my everyday professional life. | + | SC |
| 29 | I consciously chose a job where I can contribute my expertise. | + | SC |
| 30 | Almost anyone can do my work without much training. | - | SC |

*Note*. CA = creative tasks. CT = challenging tasks. PT = (pro)social tasks. CP = tasks that contribute to society. SC = tasks related to social status.

*Utrecht Management Of Identity Scale (UMICS; Crocetti et al., 2010)*

Instruction: In the following, we will focus on the personal relevance of your work in your life. For each statement, please indicate the extent to which it applies to you personally.

Response scale: 6-point Likert scale from “strongly disagree” to “strongly agree”.

**Table 4**

*Items of the UMICS Scale*

| **No.** | **Item** | **Polarity** | **Scale** |
| --- | --- | --- | --- |
| 1 | My job gives me security in life. | + | CM |
| 2 | My job gives me self-confidence. | + | CM |
| 3 | My job makes me feel sure of myself. | + | CM |
| 4 | My job gives me security for the future. | + | CM |
| 5 | My job allows me to face the future with optimism. | + | CM |
| 6 | I try to find out a lot about my job. | + | EX |
| 7 | I often reflect on my job. | + | EX |
| 8 | I make a lot of effort to keep finding out new things about my job. | + | EX |
| 9 | I often try to find out what other people think about my job. | + | EX |
| 10 | I often talk with other people about my job. | + | EX |
| 11 | I often think it would be better to try to find a different job. | + | RC |
| 12 | I often think that a different job would make my life more interesting | + | RC |
| 13 | In fact, I’m looking for a different education. | + | RC |

*Note*. CM = commitment. EX = in-depth exploration. RC = reconsideration of commitment.

*Role-based Identity Scale (RBIS; Welbourne, 2012)*

Instruction: Using the given scale, please rate how important the following aspects are to you.

Response scale: 6-point Likert scale from “not important at all” to “very important”.

**Table 5**

*Items of the RBIS Scale*

| **No.** | **Item** | **Polarity** | **Scale** |
| --- | --- | --- | --- |
| 1 | Reading journals or books associated with my profession. | + | CI |
| 2 | Having time to really think about my career. | + | CI |
| 3 | Doing things that will help me in my career. | + | CI |
| 4 | Being involved in programs that allow me to talk to others in my field. | + | CI |
| 5 | Trying out new ideas and approaches to problems. | + | EI |
| 6 | Being able to change the way things are done. | + | EI |
| 7 | Working on complex problems. | + | EI |
| 8 | Being able to change things so they’re better. | + | EI |
| 9 | Doing things that involve working with the people I work with now. | + | TI |
| 10 | Spending time with the people in my work group. | + | TI |
| 11 | Staying in a position that lets me be with my work group members. | + | TI |
| 12 | Staying with the group that I am currently working with. | + | TI |
| 13 | Being able to talk about my job with friends. | + | JI |
| 14 | Telling my family and friends about my job. | + | JI |
| 15 | Staying in the job that I have now. | + | JI |
| 16 | Being employed in my current job. | + | JI |
| 17 | Being part of the company. | + | OI |
| 18 | Working for the company. | + | OI |
| 19 | Being proud of the company. | + | OI |
| 20 | Being loyal to the company. | + | OI |

*Note*. CI = career identity. EI = entrepreneurial identity. TI = team identity. JI = job identity. OI = organizational identity.

1. Perception of the workplace

*General and specific job satisfaction (Hackman & Oldham, 1974)*

Instruction: Please rate how satisfied you are with the following aspects of your job.

Response scale: 6-point Likert scale from “strongly disagree” to “strongly agree” for Items 1-3 and a 6-point Likert scale from “not satisfied at all” to “extremely satisfied” for Items 4-8.

**Table 6**

*Items for assessing job satisfaction*

| **No.** | **Item** | **Polarity** | **Scale** |
| --- | --- | --- | --- |
| 1 | In general, I am very satisfied with my work. | + | GS |
| 2 | I often think about quitting my job. | - | GS |
| 3 | I am generally satisfied with the nature of my job. | + | GS |
| 4 | This is how satisfied I am with the amount of job security. | + | SS |
| 5 | This is how satisfied I am with the amount of personal growth and development I get in doing my job. | + | SS |
| 6 | This is how satisfied I am with the degree to which I am fairly paid for what I contribute to this organization. | + | SS |
| 7 | This is how satisfied I am with the prospects in my company (e.g.: with the security of your job in the future development of the company). | + | SS |
| 8 | This is how satisfied I am with the extent to which my job challenges me personally. | + | SS |

*Note*. GS = general job satisfaction. SS = specific job satisfaction.

*Flow Frequency Scale (FFS; Bartzik et al., 2021)*

Instruction: In the last two weeks, how often did you experience that...?

Response scale: 6-point Likert scale from “never” to “(almost) always”.

**Table 7**

*Items for assessing flow*

| **No.** | **Item** | **Polarity** |
| --- | --- | --- |
| 1 | ... you felt joy in what you were doing? | + |
| 2 | ... you were completely absorbed in your work? | - |
| 3 | ... you were challenged in just the right degree? | + |
| 4 | ... you could enjoy the work? | + |
| 5 | ... you could use your skills to the optimal extent? | + |
| 6 | ... the requirements were as made for you? | + |
| 7 | ... the work felt good? | + |
| 8 | ... you were completely focused on what you were doing? | + |
| 9 | ... you thought of nothing else during your activity? | + |
| 10 | ... all the experiences described above occurred at the same moment, i.e., you were completely absorbed in an activity and could concentrate effortlessly, while you were optimally challenged and your doing felt good? | + |

*General Self-Efficacy Short Scale (ASKU; Beierlein et al., 2012)*

Instruction: This section is about your assessment of the extent to which you can also cope with difficult situations. For each statement, please indicate the extent to which it applies to you personally.

Response scale: 6-point Likert scale from “strongly disagree” to “strongly agree”.

**Table 8**

*Items for assessing self-efficacy*

| **No.** | **Item** | **Polarity** |
| --- | --- | --- |
| 1 | In difficult situations at work, I can rely on my abilities. | + |
| 2 | I can cope well with most problems in my work on my own. | + |
| 3 | I can usually solve strenuous and complicated tasks at my work well. | + |

*Utrecht Work Engagement Scale (UWES; Schaufeli et al., 2006)*

Instruction: The following statements are about how you feel about your work. Please indicate how often or rarely your work triggers the following emotions.

Response scale: 6-point Likert scale from “never” to “(almost) always”.

**Table 9**

*Items for assessing engagement*

| **No.** | **Item** | **Polarity** |
| --- | --- | --- |
| 1 | At my work, I feel bursting with energy. | + |
| 2 | At my job, I feel strong and vigorous. | + |
| 3 | I am enthusiastic about my job. | + |
| 4 | My job inspires me. | + |
| 5 | When I get up in the morning, I feel like going to work. | + |
| 6 | I feel happy when I am working intensely. | + |
| 7 | I am proud of the work what I do. | + |
| 8 | I am immersed in my work. | + |
| 9 | I get carried away when I am working. | + |

*General well-being (WHO, 2021)*

Instruction: The following statements are about your well-being over the last two weeks. For each statement, please mark the heading that you think best describes how you have felt over the past two weeks. In the last two weeks...

Response scale: 6-point Likert scale from “at no time” to “the whole time”.

**Table 10**

*Items for assessing general well-being*

| **No.** | **Item** | **Polarity** |
| --- | --- | --- |
| 1 | … I have felt cheerful and in good spirits. | + |
| 2 | … I have felt calm and relaxed. | + |
| 3 | … I have felt active and vigorous. | + |
| 4 | … I woke up feeling fresh and rested. | + |
| 5 | … my daily life has been filled with things that interest me. | + |

1. Evaluation of working with AI

*Human-AI teaming (HAIT)*

Instruction: How would you rate the following statements regarding your collaboration with the AI System XY (replace XY with the name of the AI to be evaluated)?

Response scale: 5-point Likert scale from “strongly disagree” to “strongly agree”.

**Table 1**

*Items for measuring human-AI teaming*

| **No.** | **Item** | **Polarity** |
| --- | --- | --- |
| 1 | When working with the AI system, I feel that the AI system relieves me of tasks that I am less inclined to do myself. | + |
| 2 | When working with the AI system, we can achieve more together than I or the AI system can alone. | + |
| 3 | The AI system takes on important complementary tasks that I would otherwise not get to due to lack of time. | + |
| 4 | When working together with the AI system, we complement each other ideally. | + |
| 5 | When working with the AI system, everyone can contribute their strengths in the best possible way. | + |
| 6 | Working with the AI system means that I can concentrate fully on the important aspects of my work. | + |
| 7 | The AI system takes over tasks that I actually enjoy and would rather do myself. | - |
| 8 | Working with the AI system leads to me only doing leftover work that actually bores me. | - |
| 9 | When working with the AI, at the end of the working day I ask myself whether I actually want to continue doing this work. | - |
| 10 | I feel like a real team when working with the AI. | + |
| 11 | Working with the AI system allows me to develop more creative solutions that I would not have come up with on my own. | + |
| 12 | Working with the AI system inspires me. | + |
| 13 | Working with the AI system makes me feel like I'm part of a real team. | + |

*Task-technology fit (TTF)*

Instruction: How would you rate the following statements regarding your collaboration with the AI System XY (replace XY with the name of the AI to be evaluated)?

Response scale: 5-point Likert scale from “strongly disagree” to “strongly agree”.

**Table 12**

*Items for measuring task-technology fit*

| **No.** | **Item** | **Polarity** |
| --- | --- | --- |
| 1 | Whoever developed this AI system really thought about how to support my tasks in a meaningful way. | + |
| 2 | It is obvious that someone who understands my work was involved in the development of this AI system. | + |
| 3 | It is obvious that someone who knows my work processes was involved in the development of this AI system. | + |
| 4 | The AI system fits very well with the tasks I do. | + |
| 5 | Working with the AI system is an enrichment for my work. | + |

*Well-being in working with the AI system*

Instruction: How would you rate the following statements regarding your collaboration with the AI System XY (replace XY with the name of the AI to be evaluated)?

Response scale: 5-point Likert scale from “strongly disagree” to “strongly agree”.

**Table 13**

*Items for measuring well-being in working with the AI system*

| **No.** | **Item** | **Polarity** |
| --- | --- | --- |
| 1 | I feel comfortable working with the AI system. | + |
| 2 | I can imagine that working with such an AI system will be fun for me in the long run. | + |
| 3 | I can imagine that working with such an AI system will help me in the long run. | + |

*Evaluation of AI*

Instruction: I perceive the support by the AI system XY (replace XY with the name of the AI to be evaluated) as...

Response scale: The answer categories change according to the following pattern:

1. 6-point Likert scale from “very bad” to “very good”,
2. 6-point Likert scale from “very foolish” to “very wise”,
3. 6-point Likert scale from “very unfavorable” to “very favorable”,
4. 6-point Likert scale from “very harmful” to “very beneficial”,
5. 6-point Likert scale from “very negative” to “very positive”.

# Test construction

## Development of the JOPI

JOPI is an instrument for psychological work analysis. It is a suitable approach for uncovering sociotechnical and work related components, in which the tasks and environment are systematically captured and evaluated, as well as their influence on employees (Schaper, 2014). Various methods can be used to determine these influencing factors, including work observations, questionnaires, interviews, or simulations (Schaper, 2014). As questionnaires are a popular method in psychology that are easy to use (Krumm et al., 2021; Moosbrugger & Kelava, 2020), and offer a standardized way of examining not only work conditions, but also people’s perception of their own job and tasks, the JOPI can be described as a tool of psychological work design, consisting of different questionnaire procedures. It aims to capture relevant work characteristics, job identity, the perception of the workplace as well as the evaluation of working with AI to fulfil the important function of work analysis to identify design and optimization potentials before, during and after AI implementation. Focusing on humans instead of AI functionality, it forms the basis for adapting technologies, their use, and the organization of work to human needs and capabilities.

To develop the JOPI, we first reviewed the current literature on work characteristics, job identity, perceptual factors, and the influence of technology under the implementation of AI in the workplace. This literature formed the theoretical basis for the development of the JOPI. Then, existing questionnaires for capturing work characteristics, job identity and perceptions were examined for relevant items. When developing new items or scales the factor-analytical construction strategy was used. In this theory-based design principle, the goal is to identify groups of items or subscales of a construct that differ from other subscales of the same construct (Jonkisz et al., 2012). The items of these subscales were derived from the assumed behavioral dimensions of the defined construct and from previous literature. After the items have been developed, they are presented to a sample of subjects. With the help of the generated results, the intended factor structure with its subscales can be statistically verified (Jonkisz et al., 2012).

## Study sample

The first deployment of the JOPI to examine preliminary psychometric evidence took place in the context of speech therapy with the AI-based assistance system ISi-Speech.

ISi-Speech is an app-based training system that uses automated speech recognition software. It is intended to be used in rehabilitation for people with speech intelligibility impairments as a complement to therapy services. ISi-Speech has an integrated feedback and motivation system that enables individual, self-regulated practice situations for patients in therapy sessions and at home. The results of the patients' own training can be tracked by sharing the practice sequences. Thus, the use of ISi-Speech can relieve the speech therapist from workload and additionally provide information about the patient's performance in home exercises (Frieg et al., 2017). However, the implementation of ISi-Speech will change the work of speech therapists. The assessment of new information can change the design of the therapy sessions and their sequence. To estimate the changes accurately and to ensure a human-centered implementation, the JOPI was first used in the context of speech-language pathology at validation stage utilizing a vignette. By using a vignette, it was possible to recruit a larger number of participants, regardless of whether they were already working with an AI in their workplace and identify speech therapists job perception and identity before actual AI use. In addition, it was also possible to test the fourth section of the JOPI for AI evaluation psychometrically and in a standardized manner. Collecting data with the JOPI using a vignette made it possible to directly capture two measurement points (before and after the AI implementation) without any time delay. Moreover, the use of a vignette allowed for psychometric testing of all scales developed.

A total amount of 197 participants, all from speech therapy, was recruited. After cleaning the data, which excluded participants with conspicuous response patterns as well as those who had not answered the control questions correctly and those who did not agree with the conditions of participation, a data set with 156 participants remained. Of these, 66 participants (93.94% females) completed the survey entirely.

# Quality criteria

## Objectivity

The objectivity of implementation can be assumed since interaction between the test person and the test leader is largely avoided, and test leader effects are thus minimized. Since the participants' answers to the JOPI items are standardized and quantitatively calculated into total scale values for the individual dimensions, the evaluation objectivity can be rated as high. To increase the objectivity of the evaluation, we recommend using statistical software such as R and RStudio.

## Reliability

To determine reliability, we calculated Cronbach’s Alpha for all scales. The results can be found in table 14.

**Table 14**

*Reliability values of the JOPI scales*

| **Section** | | **Scale** | **Range of the scale** | **α** | ***N*** |
| --- | --- | --- | --- | --- | --- |
| Work  characteristics | | |  |  |  |
|  | | Relevant work  characteristics | 1;6 | .72 | 142 |
| Job  identity | |  |  |  |  |
|  | | U-MICS | 1;6 | .85 | 135 |
|  | | RBIS | 1;6 | .83 | 122 |
|  | | TBIS | 1;6 | .81 | 120 |
| Job  Perception |  | |  |  |  |
|  | | Job satisfaction | 1;6 | .85 | 146 |
|  | | UWES | 1;6 | .94 | 137 |
|  | | FFS | 1;6 | .92 | 137 |
|  | | ASKU | 1;6 | .86 | 135 |
|  | | WHO-5 | 1;6 | .84 | 150 |
| AI  evaluation |  | |  |  |  |
|  | | Human-AI teaming | 1;5 | .90 | 85 |
|  | | Task-technology fit | 1;5 | .91 | 84 |
|  | | Wellbeing working with AI | 1;5 | .89 | 83 |
|  | | Evaluation of working with AI | 1;5 | .97 | 90 |

*Note.* U-MICS = Utrecht Management of Identity Scale. RBIS = Role based identity scale. TBIS = task-based identity scale. UWES = Utrecht Work Engagement Scale. FFS = Flow Frequency Scale. ASKU = Allgemeine Selbstwirksamkeits Kurzskala [general self-efficacy short scale]. WHO-5 = World Health Organization – Five Well-Being Index.

# Conclusion

The developed inventory JOPI reveals to be a reliable self-assessment tool for the human-centered implementation of smart technologies. This inventory is thus an important tool for organizations seeking to integrate technologies into their work processes. Due to its multiple measurement points, the JOPI not only provides a punctual result, but also a starting point for a continuous evaluation of the workplace. In this way, the JOPI assists organizations in developing a human-centered workplace, which is reasonable and necessary given the ongoing developments and forecasts of technological development.

However, more research is needed to expand the data base on the JOPI. This includes testing in other occupational groups with different AI applications. Consequently, this document is intended to expand successively with future work in this area.

References

Bartzik, M., Aust, F., & Peifer, C. (2021). Negative effects of the COVID-19 pandemic on nurses can be buffered by a sense of humor and appreciation. *BMC Nursing*, *20*(1), 257. https://doi.org/10.1186/s12912-021-00770-5

Beierlein, C., Kovaleva, A., Kemper, C. J., & Rammstedt, B. (2012). Ein Messinstrument zur Erfassung subjektiver Kompetenzerwartungen: Allgemeine Selbstwirksamkeit Kurzskala (ASKU).

Brynjolfsson, E., & McAfee, A. (2014). *The Second Machine Age: Work, Progress, and Prosperity in a Time of Brilliant Technologies*. W. W. Norton & Company.

Crocetti, E., Schwartz, S. J., Fermani, A., & Meeus, W. (2010). The Utrecht-Management of Identity Commitments Scale (U-MICS). *European Journal of Psychological Assessment*, *26*(3), 172–186. https://doi.org/10.1027/1015-5759/a000024

Frank, B., & Kluge, A. (2019). Is there one best way to support skill retention? Putting practice, testing and symbolic rehearsal to the test. *Zeitschrift Für Arbeitswissenschaft*, *73*(2), 214–228. https://doi.org/10.1007/s41449-018-00136-9

Frieg, H., Mühlhaus, J., Ritterfeld, U., & Bilda, K. (2017). *Assistive Technologien in der Dysarthrietherapie*. Schulz-Kirchner Verlag. https://doi.org/10.2443/skv-s-2017-53020170302

Hackman, J. R., & Oldham, G. R. (1974). The Job Diagnostic Survey: An Instrument for the Diagnosis of Jobs and the Evaluation of Job Redesign Projects.

Huchler, N. (2022). Komplementäre Arbeitsgestaltung. Grundrisse eines Konzepts zur Humanisierung der Arbeit mit KI. *Zeitschrift für Arbeitswissenschaft*, *76*(2), 158–175. https://doi.org/10.1007/s41449-022-00319-5

Jarrahi, M. H. (2018). Artificial intelligence and the future of work: Human-AI symbiosis in organizational decision making. *Business Horizons*, *61*(4), 577–586. https://doi.org/10.1016/j.bushor.2018.03.007

Jonkisz, E., Moosbrugger, H., & Brandt, H. (2012). Planung und Entwicklung von Tests und Fragebogen. In H. Moosbrugger & A. Kelava (Eds.), *Springer-Lehrbuch. Testtheorie und Fragebogenkonstruktion* (pp. 27–74). Springer Berlin Heidelberg. https://doi.org/10.1007/978-3-642-20072-4_3

Kluge, A., Ontrup, G., Langholf, V., & Wilkens, U. (2021). Mensch-KI-Teaming: Mensch und Künstliche Intelligenz in der Arbeitswelt von morgen. *Zeitschrift Für Wirtschaftlichen Fabrikbetrieb*, *116*(10), 728–734. https://doi.org/10.1515/zwf-2021-0112

Kopp, R., Howaldt, J., & Schultze, J. (2016). Why Industry 4.0 needs Workplace Innovation: a critical look at the German debate on advanced manufacturing. *European Journal of Workplace Innovation*, *2*(1). https://doi.org/10.46364/ejwi.v2i1.373

Krumm, S., Schmidt-Atzert, L., & Amelang, M. (2021). Diagnostik in der Arbeits-, Organisations- und Wirtschaftspsychologie. In L. Schmidt-Atzert, S. Krumm, & M. Amelang (Eds.), *Psychologische Diagnostik* (6th ed., pp. 567–642). Springer. https://doi.org/10.1007/978-3-662-61643-7_6

Moosbrugger, H., & Kelava, A. (2020). *Testtheorie und Fragebogenkonstruktion*. Springer Berlin Heidelberg. https://doi.org/10.1007/978-3-662-61532-4

Parasuraman, R., Molloy, R., & Singh, I. L. (1993). Performance Consequences of Automation-Induced 'Complacency'. *The International Journal of Aviation Psychology*, *3*(1), 1–23. https://doi.org/10.1207/s15327108ijap0301_1

Schaper, N. (2014). Arbeitsanalyse und -bewertung. In F. W. Nerdinger, G. Blickle, & N. Schaper (Eds.), *Springer-Lehrbuch. Arbeits- und Organisationspsychologie* (pp. 347–370). Springer Berlin Heidelberg. https://doi.org/10.1007/978-3-642-41130-4_21

Schaufeli, W. B., Bakker, A. B., & Salanova, M. (2006). The Measurement of Work Engagement With a Short Questionnaire. *Educational and Psychological Measurement*, *66*(4), 701–716. https://doi.org/10.1177/0013164405282471

Tausch, A., & Peifer, C. (2018). Switch with the flow–Flow-Erlebnisse als Ressourcen im Schaltanlagenbau. *Positive Psychologie Und Ressourcen*, *72*.

Welbourne, T. M. (2012). The role-based identity scale: Towards a parsimonious measure of work-related identity.

World Health Organization. (2021). *Wellbeing measures in primary health care/the DepCare Project: Report on a WHO meeting: Stockholm, Sweden, 12–13 February 1998* (WHO/EURO:1998-4234-43993-62027).

Yang, N. Y., & Choi, J. S. (2014). Relationships of Nurses' Perception, Nursing Performance, Job Stress, and Burnout in Relation to the Joint Commission International Hospital Accreditation. *Journal of Korean Academy of Nursing Administration*, *20*(1), 1. https://doi.org/10.11111/jkana.2014.20.1.1
